# Synthase-selected sorting approach identifies a beta-lactone synthase in a nudibranch symbiotic bacterium

**DOI:** 10.1186/s40168-023-01560-8

**Published:** 2023-06-13

**Authors:** Mária Džunková, James J. La Clair, Tomáš Tyml, Devin Doud, Frederik Schulz, Samuel Piquer-Esteban, Dafne Porcel Sanchis, Andrew Osborn, David Robinson, Katherine B. Louie, Ben P. Bowen, Robert M. Bowers, Janey Lee, Vicente Arnau, Wladimiro Díaz-Villanueva, Ramunas Stepanauskas, Terrence Gosliner, Shailesh V. Date, Trent R. Northen, Jan-Fang Cheng, Michael D. Burkart, Tanja Woyke

**Affiliations:** 1 Department of Energy Joint Genome Institute, Berkeley, CA USA; 2 Environmental Genomics and Systems Biology, Lawrence Berkeley National Laboratory, Berkeley, CA USA; 3 Institute for Integrative Systems Biology, University of Valencia and Consejo Superior de Investigaciones Científicas (CSIC), Valencia, Spain; 4 Department of Chemistry and Biochemistry, University of California, San Diego, CA USA; 5 Molecular Biophysics & Integrated Bioimaging, Lawrence Berkeley National Laboratory, Berkeley, CA USA; 6 Laboratory for Research in Complex Systems, Menlo Park, CA USA; 7 Foundation for the Promotion of Sanitary and Biomedical Research of the Valencian Community (FISABIO), Valencia, Spain; 8 Bigelow Laboratory for Ocean Sciences, East Boothbay, ME USA; 9 California Academy of Sciences, San Francisco, CA USA; 10 University of California San Francisco, San Francisco, CA USA; 11 San Francisco State University, San Francisco, CA USA; 12 University of California Merced, Life and Environmental Sciences, Merced, CA USA

**Keywords:** Microbial single-cell genomics, Nudibranchs, Tethybacterales, Beta-lactone, Biosynthetic gene clusters

## Abstract

**Background:**

Nudibranchs comprise a group of > 6000 marine soft-bodied mollusk species known to use secondary metabolites (natural products) for chemical defense. The full diversity of these metabolites and whether symbiotic microbes are responsible for their synthesis remains unexplored. Another issue in searching for undiscovered natural products is that computational analysis of genomes of uncultured microbes can result in detection of novel biosynthetic gene clusters; however, their in vivo functionality is not guaranteed which limits further exploration of their pharmaceutical or industrial potential. To overcome these challenges, we used a fluorescent pantetheine probe, which produces a fluorescent CoA-analog employed in biosynthesis of secondary metabolites, to label and capture bacterial symbionts actively producing these compounds in the mantle of the nudibranch *Doriopsilla fulva.*

**Results:**

We recovered the genome of *Candidatus* Doriopsillibacter californiensis from the *Ca.* Tethybacterales order, an uncultured lineage of sponge symbionts not found in nudibranchs previously. It forms part of the core skin microbiome of *D. fulva* and is nearly absent in its internal organs. We showed that crude extracts of *D. fulva* contained secondary metabolites that were consistent with the presence of a beta-lactone encoded in *Ca.* D. californiensis genome. Beta-lactones represent an underexplored group of secondary metabolites with pharmaceutical potential that have not been reported in nudibranchs previously.

**Conclusions:**

Altogether, this study shows how probe-based, targeted sorting approaches can capture bacterial symbionts producing secondary metabolites in vivo.

Video Abstract

**Supplementary Information:**

The online version contains supplementary material available at 10.1186/s40168-023-01560-8.

## Background

Due to increased incidence of severe and untreatable diseases, the emergence of public health threats, as well as growing pesticide and insecticide resistances in agriculture, there is a continuous scientific effort to gain access to natural products with unprecedented structures [1]. However, large screening programs show that if secondary metabolites are explored by common approaches and use easy-to-reach samples, such as soil or plants, the same compounds are often re-discovered [2]. Therefore, research efforts in recent years have focused on underexplored sources, such as the microbiomes of marine animals [3].

Soft-bodied marine animals, including sponges, tunicates, and nudibranchs, are known to use secondary metabolites as a protective strategy to fend off their potential predators [4]. Currently, there are 20 drugs from marine animals approved for clinical use, e.g., anticancer drugs Cytarabine, Ecteinascidin, Eribulin, Brentuximab, antiviral Vidarabine, and analgesic Ziconotide [5]. Most of the secondary metabolites from marine animals have been discovered directly from tissue extracts; however, the presence of these compounds in animal tissues does not automatically mean that they are produced by the animals themselves [6]. Natural products detected in marine animals can accumulate through the food web [7] or be synthesized by symbiotically associated bacteria [8–10]. Nevertheless, the difficulty in culturing symbiotic bacteria has limited our ability to study these natural products using traditional cultivation-based approaches [11, 12]. Fortunately, computational tools for identification of biosynthetic gene clusters (BGCs) in (meta)genomic assemblies developed in the last decade can elucidate the microbial origin of some natural products [13, 14]. For example, metagenomic sequencing recently demonstrated that kahalalides isolated from the marine slug *Elysia rufescens* are not produced by the *Bryopsis* algae it feeds on, but by symbiotic microbes of this algae [7]. Recent surveys of thousands of microbial genomes recovered from single cells [15] and metagenomes [16] in the global ocean indicated the presence of an enormous diversity of BGCs in free-living or host-associated marine bacteria. However, it is unclear which of the thousands of BGCs from the uncultured microbes are most suitable for further biochemical characterization.

Secondary metabolites produced by BGCs in uncultured bacteria are usually biochemically characterized in culturable heterologous hosts [17]. However, the successful expression of new secondary metabolites is limited to molecular groups with well-characterized biosynthetic pathways [18]. For example, decades of biochemical studies enabled the detection of BGCs for the synthesis of polybrominated diphenyl ethers in uncultured cyanobacterial endosymbionts of marine sponges, and subsequent mass spectrometry of these BGCs expressed by heterologous hosts revealed new structures [10]. In contrast, BGCs of underexplored molecular groups can be predicted by computational tools, but their in vivo functionality is not guaranteed [19]. For example, the predicted BGCs could have undergone mutational events that prevent biosynthesis, such as active site mutations, loss of key domain structures, or ablation of protein–protein interactions that are critical to the macromolecular protein assembly that guides the biosynthetic process [20]. To avoid wasting resources on attempts to synthesize secondary metabolites from underexplored molecular groups in heterologous hosts, it is necessary to ensure that the predicted BGCs are functional in the native bacterial cells.

In the present study, we used an activity-guided cell sorting approach [21] to detect, characterize, recover, and confirm expression of BGCs harbored in genomes of bacterial symbionts of nudibranchs. Nudibranchs are known to contain a variety of toxins [22–24]; however, to date, only a small subset of the 6000 nudibranch species have been explored [25]. Our understanding of symbionts associated with nudibranchs is limited to microscopical observation [26–28], 16S rDNA amplicon sequencing of uncultured bacteria [29, 30], PCR screening of culture isolates by universal non-ribosomal peptide synthetase (NRPS) or polyketide synthase (PKS) primers, and bioactivity testing of their microbial culture extracts [9, 31–34]. To the best of our knowledge, no metagenome-amplified genomes (MAGs) or single-amplified genomes (SAGs) of uncultured microbes from nudibranch microbiomes have been sequenced. In comparison, there are hundreds of MAGs and SAGs from sponges and corals, and many of them were found to harbor undiscovered BGCs by computational algorithms; however, further investigation is often hampered, leaving uncertainty about their functionality [35–45].

**Fig. 1:**
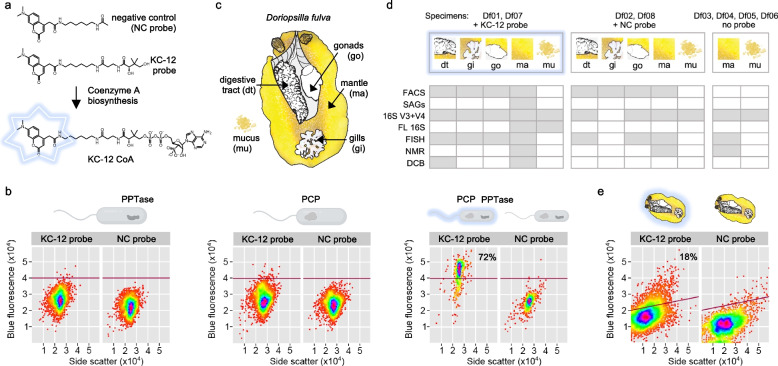
Overview of the study design. **a** Molecular structure of the NC probe (negative control) and KC-12 probe and attachment of KC-12 to acetyl-CoA leading to KC-12 CoA. **b** Flow cytometry bi-plots showing blue fluorescence produced by KC-12 probe on the *y*-axis vs. side scatter on the *x*-axis for *E. coli* cells possessing plasmids with/without phosphopantetheinyl transferase (PPTase) and a compatible carrier protein (PCP). The *E. coli* strains with either PCP or PPTase did not produce any fluorescence (1st and 2nd plot), while the strain possessing both PCP and PPTase was fluorescent when treated with KC-12 probe (3rd plot). **c** Nudibranch *Doriopsilla fulva.* **d** Summary of methods that were applied, indicated by gray boxes, to different body parts of nudibranchs incubated with KC-12 probe, NC probe, or not incubated with any probe. **e** Flow cytometry bi-plots showing blue fluorescence produced by KC-12 probe on *y*-axis vs. side scatter on *x*-axis for *D. fulva* skin microbiome samples (Df01 and Df02) incubated with the KC-12 probe and NC control. Incubation with KC-12 resulted in staining of 18% of the viable cells. Flow cytometry axes are on log scale

To detect nudibranch-associated bacteria actively producing secondary metabolites encoded by functional BGCs, we used a fluorescently labeled analog of pantetheine (probe KC-12, Fig. 1a). We have previously shown that the probe KC-12 hijacks coenzyme A (CoA) biosynthesis in cells to produce a fluorescently-labeled analog of coenzyme A (Fig. 1a) [46, 47]. This fluorescently labeled CoA can be post-translationally added to the active site serine residue on acyl-carrier proteins (ACP) and peptidyl-carrier proteins (PCPs) associated with PKS and NRPS, respectively [46, 47]. The uptake of the KC-12 probe in bacterial cells undergoes a two-step process beginning with conversion to its corresponding CoA and then covalent 4’-phosphopantetheinylation of the active site serine residue on ACP and PCP-containing proteins with the modified CoA. This ACP/PCP protein-labeling event was used in this study to indicate cells that have an increased level of polyketide or non-ribosomal peptide activity. We employed this PKS/NRPS-biosynthetic fluorescent marker system in a fluorescence activated cell sorting (FACS) assay, followed by cell lysis and subsequent whole genome amplification and sequencing [48] of bacteria from mantle microbiome of the nudibranch *Doriopsilla fulva* [49]. We discovered the first secondary metabolite produced by symbiotic microbes of nudibranchs.

## Methods

### Benchmarking targeted single-cell screening approach for capturing bacteria actively producing secondary metabolites in vivo

The fluorescent pantetheine probe (KC-12) and the negative control (NC) lacking the terminal pantoic amide were synthesized as previously reported [50]. Samples of this probe were purified to > 99% purity by preparative thin layer chromatography developing with 5:1 CH_2_Cl_2_: MeOH and eluting with 2:1 CH_2_Cl_2_: MeOH. The resulting material was aliquoted at 1 mg into ½ dram vials and stored dry at 0 °C until needed.

To establish specificity of the ACP/PCP-labeling KC-12 probe (Fig. 1a), we developed a flow cytometry assay to screen a culture of engineered *Escherichia coli* BL21 (New England Biolabs), which contained plasmids expressing components necessary for NRP synthesis, including a peptidyl carrier protein domain (PCP) of NRPS [50] and 4’-phosphopantetheinyl transferase (PPTase) [51]. Two additional strains of *E. coli* containing the individual plasmids expressing either PCP or PPTase were used as negative controls. The specificity of the system was confirmed by a negative control (NC) probe (Fig. 1a) which contained the same fluorescent moiety but lacked the terminal pantoic amide critical for PPTase loading on the PCP. The strains were cultured at 37ºC in LB overnight with addition of 1 mM IPTG or no IPTG. For labeling, 250 μM of KC probe, 250 μM of NC probe, or no probe were used. Afterwards, the strains were inspected by BD Influx™ system (BD Biosciences, San Jose, USA) on a bi-plot of forward scatter vs. blue fluorescence (435–485 nm). As a result, the KC-12 probe stained only the *E. coli* strain expressing both the PCP and PPTase (Fig. 1b). In addition, we performed KC-12 labeling of *Photorhabdus luminescens* naturally producing a variety of secondary metabolites, which after reaching the exponential growth phase showed a stable ratio of fluorescent cells (Supplementary Fig. S1). Using the treatment conditions developed in this benchmark study, we next embarked on a probe-based in vivo labeling and sorting experiment in live nudibranchs collected in the field.

### Sampling, labeling, dissection, and identification of nudibranch host species

In total, eight *Doriopsilla fulva* (*Dendrodorididae*, Fig. 1c) nudibranchs (Df01-08) were collected at the Pillar Point tide pools (37º 29ʹ 41.427″ N, 122º 29ʹ 57.994″ W) between June 2017 and July 2021 (Supplementary Table S1). Approval for collection of nudibranchs was granted by the State of California—Department of Fish and Wildlife (Specific use permits D-0019083377–8 and S-202590005–20,259-001). During the collecting trips, all specimens were identified, based on their morphology, as *Doriopsilla fulva* (Dendrodorididae). The species identification was further confirmed by Sanger sequencing of amplicons of the mitochondrial cytochrome C oxidase subunit I [52] and histone H3 [53], as described below for sequencing on Applied Biosystems 3730XL DNA Analyzer at UC Berkeley sequencing core facility. Primers HCO2198 5′-TAA ACT TCA GGG TGA CCA AAA AAT CA-3′ and LCO1490 5′-GGT CAA CAA ATC ATA AAG ATA TTGG-3′ were employed in a 25 µl volume PCR reaction using the KAPA HiFi DNA Polymerase kit (KK2102, KAPA) under the following conditions: 95ºC 3 min, 35 cycles of: 95ºC 30 s, 40ºC for 60 s, and 72ºC for 90 s, followed by 72ºC for 2 min. The primers for histone H3 amplification H3-AF 5′-ATG GCT CGT ACC AAG CAG ACG GC-3′ and H3-AR 5′-ATA TCC TTG GGC ATG ATG GTG AC-3′ were used in the following PCR conditions: 95ºC 2 min, 35 cycles of 95ºC 30 s, 55ºC for 60 s, and 72ºC for 75 s, followed by 72ºC for 2 min.

All specimens were transported to the laboratory (2-h drive) while alive and in a cooler box to avoid a steep temperature increase. Nudibranchs were either euthanized by a scalpel blade and dissected immediately after arrival at the laboratory or kept in filtered seawater from the sampling locality supplemented with either 250 µM KC-12 or 250 µM NC probe (Fig. 1d). All incubation experiments were performed at 15ºC overnight. Immediately following euthanasia, mucus was scraped off intact mantle skin and subsequently mantle, gills, and internal organs (hepatopancreas, pharynx, stomach, digestive glands, intestine, caecum, gonads) were dissected and split for further processing (sorting, DNA and RNA extraction, histology, beta-lactone extraction).

### FACS of nudibranch microbiome and bacterial genome sequencing

The first two collected *D. fulva* nudibranchs Df01 and Df02 (Fig. 1c, d, Supplementary Table S1) were incubated either with 250 µM of KC-12 probe (Df01) or 250 µM of NC probe (Df02), their body organs were dissected and disrupted with a tissue homogenizer, and the homogenates were then filtered through 5-µm syringe filters. The resulting filtrates were stained with SYTO61 (a generic red fluorescent nucleic dye for viable cells) or left unstained before being analyzed by BD Influx™ FACS system (BD Biosciences, San Jose, USA) with a 70-μm nozzle, using a sheath fluid consisting of 1X PBS, which was treated overnight by UV irradiation. The fluidic lines were sterilized before sorting by flowing through a 10% bleach solution for 2 h. The samples were visualized by forward scatter vs. red fluorescence (650–690 nm) bi-plots to set the first gate marking live cells (stained with SYTO 61). Gated events were then visualized on a bi-plot of side scatter vs. blue fluorescence (435–485 nm) to capture cells that acquired the pantetheine probe KC-12, which were identified by comparison with the sample labeled with the NC probe. Nudibranch microbiome cells targeted for genome sequencing were sorted into 384 well plates as single cells (*n* = 24) or as bulks of 5 (*n* = 24), 10 (*n* = 24), 25 (*n* = 24), 50 (*n* = 24), or 100 cells (*n* = 12) and lysed with a combination of freeze-thawing and alkaline lysis and amplified with the REPLI-g Single-cell kit (150,343, Qiagen) applied by Echo® 550 liquid handling system (Labcyte, Sunnyvale, CA) as described previously [54, 55]. Samples that were successfully amplified were then processed using Nextera XT (Illumina), and the sequencing was performed on the Illumina NextSeq platform in 2 × 150 bp mode.

### Sequence read processing, assembly, and binning

Raw reads were filtered for quality and contamination with BBDuk from the BBTools v.38.69 [56] package, then BBTools components BBNorm and Tadpole were used for read normalization and error correction. Afterwards, the reads were assembled with SPAdes v3.13.0 using parameters –phred-offset 33 –sc -k 22, 55, 95 [57]. According to the IMG standard protocols, 200 bp were trimmed from each contig end, and contigs < 2 kbp or with read coverage < 2 were discarded [58]. CheckM v1.0.13 with a lineage-specific workflow [59] was used to estimate completeness and only the assemblies with genome completeness > 10% were used for the following analysis. Assemblies were binned using MetaBAT2 v2.12.1 [60]. Samples containing bins belonging to the same bacterial species (sequence similarities > 99% on more than 95% of their total assembly length as detected by Mash v1.1 [61]) were co-assembled with SPAdes v3.13.0. In order to close the gaps between the contigs in the resulting co-assembly, we took the original individual assemblies used for this co-assembly and searched for sequences with > 99% sequence similarity to the ends of the contigs in the co-assembly.

### Strain-level diversity analysis

Reads from the separate assemblies were mapped to the final co-assembly with BBMap v38.58 [56], and the presence of single-nucleotide polymorphism (SNP) and insertions or deletions (indels) was assessed by VarScan v2.3.9 [62]. Positions with read depth coverage of > 30 and variance in > 90% of mapped reads were considered.

### Genome-based phylogenomics and metabolic predictions

All individual assemblies and the co-assembly were analyzed together with a representative set of bacteria and archaea based on all publicly available microbial genomes in IMG/M [58] (genomes accessed in May 2020). The phylogenetic tree was built using a set of 56 universal single copy marker proteins [63, 64], which were identified with hmmsearch v3.1b2 [65], using a specific HMM for each of the markers. Alignments for each protein marker were built with MAFFT v7.294b [66] and subsequently trimmed with BMGE using BLOSUM30 [67]. Single-protein alignments were then concatenated, resulting in an alignment of 10,866 sites. Maximum likelihood phylogenies were inferred with FastTree2 using the options: -spr 4 -mlacc 2 -slownni –lg [68] to obtain initial taxonomic classification. The final phylogenetic tree contained representatives of all families of the matched phylum (Proteobacteria) and all medium quality genomes of the matched order (*Ca.* Tethybacterales [38, 39], see the “Results” section). It was built as described above except that phylogenetic inference was performed in iq-tree v2.0.3 [69] using the LG4X + F model. The AAI between the assemblies and all other previously published members of *Ca.* Tethybacterales was calculated via the enveomics online tool [70] considering genus and family thresholds established by Konstantinidis et al*.* [71]. For the herein discovered nudibranch symbiont, we proposed the name *Candidatus* Doriopsillibacter californiensis acknowledging its host genus and the geographic location of discovery (Supplementary Note 1). The metabolic potential of *Ca.* D. californiensis and other members of *Ca.* Tethybacterales was analyzed with the KEGG search tool BlastKOALA v2.2 [72].

### Detection of *Ca*. D. californiensis relatives in public databases and 16S rDNA based phylogeny

To explore the hidden diversity of bacterial groups related to *Ca. D. californiensis*, we searched for contigs that were not binned as MAGs by the IMG pipeline. All proteins found in the *Ca. D. californiensis* genome were compared by blastp (> 70% similarity on 30% of the alignment length) to all IMG/M assemblies, which also included unbinned sequences. Matched contigs were then compared to the NCBI “nr” database to verify which bacterial species had best hit was to the *Ca.* D. californiensis genome.

Further, 16S rDNA sequences extracted from the *Ca.* D. californiensis genome were compared to the IMNGS database containing data from, at the time of analysis (October 2020), 422,877 amplicon sequencing runs from the Sequence Read Archive (SRA) [73]. In addition, we used 16S rDNA amplicon sequences from the nudibranch microbiome studies of Cleary et al. [29] and Abdelrahman et al. [30], which were not included in the IMNGS at the time of the analysis. From the three datasets, we kept only sequences with similarity > 92%, which corresponds to the family threshold for full length 16S rDNA sequences according to Yarza et al. [74]. These sequences were clustered at a 99% similarity level by usearch v11.0.667 [75] and used to build a phylogenetic tree, along with the full length or nearly full length 16S rDNA sequences from the *Ca.* Tethybacterales order from the studies of Taylor et al. [38] and Waterworth et al. [39]. The sequences were aligned with cmalign Infernal v.1.1.2 [76] using the Rfam model for the 16S rRNA gene (RF00177), and the phylogenies were inferred with iq-tree v2.0.3 [69] using the TIM3e + R7 model, which was selected as the best fit model based on the Bayesian information criterion.

### Full-length 16S rDNA from nudibranch mantle and mucus and 16S rDNA-based phylogeny

A 20-µl aliquot of the homogenized 5-µm-filtered mantle tissues and mucus collected separately from nudibranch Df01 were used for DNA extraction by alkaline lysis, which simulates DNA extraction conditions used for FACS-sorted cells. Briefly, the filtered samples were combined with a 14-µl lysis buffer (prepared by combination of 700 µl KOH stock 0.43 g/10 ml, 430 µl DDT stock 0.8 g/10 ml, and 370 µl water, pH adjusted to 12). The tubes were vortexed and incubated at room temperature for 10 min and then kept at − 80ºC for 1 h. Immediately afterwards, the samples were placed into a heat block set to 55ºC for 5 min. The reaction was neutralized by adding 14 µl stop buffer (0.5 g/ml Tris–HCl, pH adjusted to 4) and vortexing. The DNA was purified with 86.4 µl AMPure XP beads (A63881, Beckman Coulter) according to the manufacturer’s instructions.

The full-length 16S rRNA gene was amplified from the extracted DNA with 8F primer 5′-AGA GTT TGA TCA TGG CTC AG-3′ and 1509R primer 5′-GGT TAC CTT GTT ACG ACT T-3′ [77] using Taq DNA polymerase (10,342,053, Fisher) in a 25-µl reaction volume including 3-µl extracted DNA and conditions: 95 °C for 3 min, 30 cycles of 95 °C for 30 s, 50 °C for 30 s, and 72 °C for 90 s, followed by 72 °C for 5 min. The PCR products were then excised from 1.2% agarose gel and purified with High Pure PCR Product Purification Kit (11,732,668,001, Sigma-Aldrich). The resulting 19.2 ng of purified PCR product was cloned into pCR 2.1 vector using TA Cloning Kit (K2020-20, Fisher) and transformed into One Shot OmniMAX 2 T1R chemically competent *E. coli* cells for blue-white screening (C854003, Fisher). The presence of the insert was confirmed by colony PCR using KAPA HiFi DNA Polymerase kit (KK2102, KAPA) targeting M13 alignment sites of the vector following manufacturer’s instructions. The PCR reactions were purified by PCR Cleanup beads and sequenced on an Applied Biosystems 3730XL DNA Analyzer at the UC Berkeley sequencing core facility. To obtain full-length 16S rDNA sequences, the forward and reverse sequences were merged. A total of 400 clones (200 from each sample) out of 500 were successfully sequenced.

A phylogenetic tree was built with Silva Alignment, Classification, and Tree Service v1.2.11 [78] using the RAxML method [79]. All the phylogenetic trees in this study were visualized by iTOL v6 [80].

### Assessment of the presence of *Ca.* D. californiensis by semi-quantitative qPCR

DNA was extracted from different body organs from 7 nudibranchs (Supplementary Table S1) using alkaline lysis as described above, along with a negative control for DNA extraction (water). We also analyzed the DNA extracted from a seawater sample (200 ml) collected at the same sampling site on the 24th of June 2021 (when no nudibranch was collected) and the DNA extracted from soil near Hopland, CA. In addition, we included seawater samples (200 ml) from the container in which nudibranchs were transported from the sampling site to the laboratory (about 2 h), and samples of sterile water, in which nudibranch were incubated with the KC-12 probe, with some residues of nudibranch mucus present in this sample.

Full-length 16S rDNA was first amplified in a primary PCR using universal primers to obtain full-length 16S rDNA amplicons (8F primer 5′-AGA GTT TGA TCM TGG CTC AG-3′ and 1509R primers 5′-GGT TAC CTT GTT ACG ACTT-3′ [77]). A 25-µl volume PCR reaction was performed using the KAPA HiFi DNA Polymerase kit (KK2102, KAPA) under the following conditions: 95ºC 3 min, 23 cycles of 95ºC 30 s, 50ºC for 30 s, and 72ºC for 90 s, followed by 72ºC for 5 min. The PCR amplicons were purified using PCR clean-up magnetic beads (UC Berkeley DNA sequencing facility) and normalized to a concentration of 1 ng/μl. The correct amplicon length was confirmed by electrophoresis on an 0.8% agarose gel. Samples from body organs, which did not reach the correct concentration of amplicons after three extraction attempts, were excluded from the following experiments.

The resulting purified amplicons were amplified with *Ca.* D. californiensis-specific primers Dc-16S-447-F: 5′-CTT TGC CGC TCT CAA TTA TGG-3′ and Dc-16S-1436-R 5′-TCA AAT TGG GCG TTC CCT CTT-3′ in a secondary PCR using the KAPA SYBR Fast kit (KK4611, KAPA) in 12.5 μl reactions analyzed on LightCycler 480 (Roche) using the following amplification program: 95ºC 3 min, 38 cycles of 95ºC 30 s, 50ºC for 30 s, and 72ºC for 60 s, followed by 72ºC for 5 min and the melting curve analysis. A standard curve was constructed using samples containing serial dilutions of the full length 16S rDNA clones obtained in the Sanger sequencing step described above. The quantification was performed in three technical replicates, starting from three primary PCR replicates from each DNA extraction. Only the samples which matched the melting curve profile of the positive control were considered positive.

### Analysis of the 16S rDNA V3-V4 regions from nudibranch body organs

The same samples used for the nested PCR described above were used for amplification of the V3 and V4 regions of the 16S rDNA using primers 341F: 5′-GCT CTT CCG ATCT -N- CCT ACG GGN GGC WGC AG-3′ and 805R: 5′-GCT CTT CCG ATCT -N- GAC TAC HVG GGT ATC TAA TCC-3′, with staggering diversity of 1–5 Ns placed between the Illumina overhang and the primer sequence. The DNA was amplified in triplicates using the KAPA HiFi DNA Polymerase kit (KK2102, KAPA) under the following conditions: 95ºC 3 min, 28 cycles of 95ºC 30 s, 55ºC for 30 s, and 72ºC for 30 s, followed by 72ºC for 5 min. The amplicons were purified with PCR clean-up magnetic beads (UC Berkeley DNA sequencing facility), indexed, and prepared for sequencing on Illumina MiSeq 300PE v3 at the QB3 Genomics at UC Berkeley. The sequencing produced an average of 121,002 ± 51,926 reads per sample. One of the triplicates of the Df07 mucus was excluded from further analyses due to a low number of reads (570 reads).

Illumina adapters were removed using Fastp v.0.23.2 [81] with parameters -detect_adapter_for_pe, -disable_quality_filtering and -disable_length_filtering. Primers 341F and 805R, found in these sequencing runs in mixed opposite orientations, were removed by Cutadapt v3.5 [82], using the linked behavior, an overlap of 10 nucleotides, removing reads of length zero and discarding untrimmed sequences, which resulted in four sequencing files per sample. Sequences were further deduplicated with the filterbyname.sh script of the BBTools suite version 38.95 [56]; sequences were then processed as separate pairs for each orientation. The resulting reads were processed with the DADA2 pipeline v1.22.0 [83]. In brief, R1 and R2 reads were truncated at 260 bp and 220 bp, and low-quality R1 and R2 reads were filtered using a max expected error of 2 and 5, respectively. Exact amplicon sequence variants (ASVs) were determined using the core sample inference algorithm of DADA2 v1.22.0 using the pool inference behavior, and pair-end reads were merged. The two resulting files with sequences in opposite orientations were unified with the reverseComplement function of the Biostrings v2.62.0 [84], and the two result tables were merged using the mergeSequenceTables function of DADA2 v1.22.0. Finally, chimeras were removed from the resulting file with DADA2 v1.22.0 using the default consensus method.

A set of 12,070 ASVs was then used in the taxonomic analysis. Our initial analyses showed that the commonly used databases, Silva [85] and RDP [86], failed to classify the members of the *Ca.* Tethybacterales order, due to absence of *Ca.* Tethybacterales in these databases and incompatibility of the taxonomy strings. The *Ca.* Tethybacterales order was first described in the study of Taylor et al*.* [38], based on the GTDB taxonomy [87]. Therefore, we decided to use the SBDI Sativa curated 16S rDNA GTDB database [88] and amend it with the *Ca.* D. californiensis full length 16S rDNA and other *Ca.* Tethybacterales sequences [38, 39], which at the time of analysis were yet not included in the GDTB. To identify eukaryotic contamination, we added to the SBDI Sativa GTDB database a set of 365 mitochondrial and 1674 chloroplast sequences from the SILVA SSU 138.1 database. This set included sequences that were 1000–2000 bp long, did not contain any undetermined bases, did match 16S rRNA gene profiles from Barrnap (https://github.com/tseemann/barrnap), and included the longest five sequences representing each taxon. Taxonomic classification was carried out employing the IDTAXA classifier [89] from the package DECIPHER v2.22 [90] using a 50% confidence threshold and default parameters. Using this approach, we could classify 71.5% of total reads to the genus level, which was 1.5 × more reads classified than with the common Silva database. After classification, ASVs identified as from organelles (mitochondria and chloroplast) were eliminated, resulting in a final set of 11,869 ASVs that was imported to the phyloseq R package v1.40.0 [91].

Differences between replicates were examined by means of a Principal Component Analysis (PCA). Lowly abundant taxa were filtered, keeping only those with a relative abundance greater than 0.0001 (0.01%) in at least one of the samples and then the abundance data were transformed using the Centered Log Ratio (CLR) transformation as implemented in the microbiome R package v1.18.0 [92]. The scores of the associated ASVs were inspected with the Vegan R package v2.6–2 [93] to investigate the main taxonomic drivers of any differences between replicates. Since no major differences were observed between the replicates of the same sample, replicates with greater read counts were retained as representatives of each sample for the rest of the analysis.

In the final step, we focused on defining the core microbiome of the mantle, to detect essential mantle symbionts. The core microbiome was defined setting a 100% prevalence threshold (prevalent in all seven mantle top replicate samples) and a 0.01% relative abundance threshold. The abundance and prevalence of the resulting core ASVs were examined employing the ComplexHeatmap R package v2.12.0 [94].

### Fluorescent in situ hybridization

The probes for fluorescent in situ hybridization (FISH) of *Ca.* D. californiensis-specific 16S rRNA were designed using Primrose v2.17 [95] aiming to obtain a sequence 19–21 nucleotides long allowing no non-target matches when compared to the RDP 16S rRNA database [86]. The following probes were selected: Dcal-447–468 5′-/Cy3/-GGT ATT AAC TCT CGC CGT TTC-3′ and Dcal-1–21 5′-/Cy3/CTG AGC CAG GAT CAA ACT CTT-3′. The probes were tested for their specificity with clones containing full-length 16S rDNA sequences belonging to *Marimonas*, *Colwellia*, *Oleispira*, *Shewanella*, and *Vibrio* (most common Gammaproteobacteria detected by Sanger sequencing in the previous step).

Immediately following euthanasia, excisions of mantle and other organs (gills, hepatopancreas, intestine, and gonads) from four *D*. *fulva* specimens (Df05-Df08) collected in 2021 (Supplementary Table S1) were fixed overnight at 4ºC in 4% buffered formaldehyde (Electron Microscopy Sciences, Hatfield, PA) diluted in filtered sea water. The tissue samples were then transferred to 70% ethanol and shipped to HistoWiz Inc. (Brooklyn, NY) for histological processing according to their standard operating procedure (paraffin embedding and sectioning). Sections of 4 µm thickness were either stained with hematoxylin and eosin and periodic acid-Schiff or mounted on silane-coated slides (Electron Microscopy Sciences, Hatfield, PA) and shipped back. For fluorescence in situ hybridization (FISH), the sections were deparaffinized in two changes of Histo-Clear II (Electron Microscopy Sciences), rehydrated in a graded ethanol series followed by 20 mM Tris/HCl buffer (pH 8.0). The rehydrated sections were incubated at 46 °C for 2–6 h with two Cy5 double-labeled *Ca.* D. californiensis-specific probes (Dcal-447–468, Dcal-1–21) along with a mix of Alexa Fluor 488-labeled EUB338 I-III probes [96] and Cy3-labeled EUK516 probe targeting the host tissues [97] in a hybridization buffer made up of 20% of formamide according to a standard protocol [98]. The optimal formamide concentration and non-specific interactions [99] were tested in a series of FISH experiments carried out on previously frozen cell suspensions prepared for FACS and repeated on histological sections. The fluorescently labeled sections were stabilized either in ProLong glass antifade mountant supplemented with the Hoechst 33,342 counterstain (ThermoFisher Scientific, Waltham, MA) or in EverBrite TrueBlack lipofuscin quenching mountant (Biotium, Fremont, CA) and examined within 24 h on epifluorescence microscope Zeiss Observer.D1 equipped with AxioCam MRm camera.

### Biosynthetic pathways detection

Individual assemblies and the co-assembly of *Ca.* D. californiensis were searched for the biosynthetic gene clusters (BGCs) by AntiSMASH v5.0.0 and v6.1.0 [100] using the default relaxed detection strictness, which resulted in detection of the *Ca.* D. californiensis beta-lactone (DCB). A set of BGCs identified as best matches to the DCB by the two AntiSMASH versions and the whole genome assemblies containing these BGCs were compared with the individual genes in the DCB cluster with blastp and tblastn, respectively, taking into account matches with > 30% AA sequence similarity on > 60% sequence length. In addition, we searched for genes with the highest AA sequence similarities to DCB using the “nr” database of NCBI. The assemblies containing the best matches were then analyzed by AntiSMASH v6.1.0 for the presence of the BGCs. The same analysis was performed with the nine medium quality MAGs from the *Ca.* Tethybacterales order.

### Extraction and localization of metabolites from nudibranch skin

We began by evaluating conditions for extraction: samples of Df03 (15 ± 5 mg) were extracted using 3 × 2 ml CH_2_Cl_2_, 3 × 2 ml acetone, or 3 × 2 ml ethyl acetate, and the combined fractions from each solvent were independently dried by airflow. The entire sample was then dissolved in 50 µl of acetone-*d*_6_, and NMR data was collected on a Bruker Avance III 600 MHz equipped with 1.7-mm inverse detection triple resonance (H-C/N/D) cryoprobe with z-gradients. The ^1^H spectra obtained from this study indicated that extraction with acetone provided the best yield. We then conducted a further extraction of the tissue remaining from the acetone extraction with methanol and obtained an additional fraction. As the MeOH fraction contained materials that were not extracted with acetone, we switched our NMR solvent from CD_3_OD. Using this evidence, we developed a protocol for tissue extraction that provided two fractions through the sequential extraction with 3 × 2 ml acetone and then 3 × 2 ml MeOH. Using this method, we were able to collect detailed NMR spectral data set from nudibranchs Df07 and Df08. Immediately after collecting this NMR data, the samples were split in half and subjected to LC-HMRS and NMR-guided purification using high-performance thin layer chromatography (HP-TLC) with the goal of collecting supportive MS data and conducting NMR-guided isolation. Unfortunately, due to the small amounts of material left at this stage, neither approach provided sufficient data (lack of peaks in the LC) and lack of NMR signals in the isolated materials. While we considered scaling up this process, the fact that *D. fulva* typically only grow to 33 mm and are not observed at high populations (our typical collection trip returned 0–3 specimens) along with concerns over ecological impact if such sampling was conducted, prevented further investigations.

### Chassis-independent recombinase-assisted genome engineering (CRAGE)

The sequence containing six core DCB biosynthetic genes was refactored to form a single operon using the BOOST design software [101] and an *E. coli* codon frequency table. Ribosome binding sites that facilitate a high translational rate (determined by the BRS Calculator) were added 5′ to each gene. The operon contained an IPTG inducible T7 promoter at the 5′ end and a T7 terminator at the 3′ end. This operon was partitioned into 3 overlapping synthetic building blocks (obtained from Twist Bioscience, CA, USA), which were later PCR amplified and assembled into the pR6K-loxWT5171 vector [102] using the NEBuilder Hi-Fi Assembly kit (E2621X, NE BioLabs). The synthetic building block and PCR primer sequences are listed in the Supplementary Table S2. The sequence of the refactored DCB operon construct pR6K-2L-DCB was verified by Pacific Bioscience sequencing. The pR6K-2L-DCB was first transformed into *E. coli* BW29427 (aka WM3064) cells, which were then used as a conjugal donor to deliver the plasmid into the 9 recipient bacteria: *Aeromonas piscicola*, *Aeromonas salmonicida subsp. pectinolytica* 34mel, *Dickeya solani*, *Erwinia oleae*, *Pantoea agglomerans*, *Pseudomonas putida* KT2440*, Serratia odorifera*, *Yersinia aldovae*, and *Yersinia mollaretii* (Supplementary Table S3). The conjugation procedure described by Liu et al. [102] was used to introduce the plasmid into the recipient cells with slightly different conjugation incubation periods depending on the growth rate of the recipient cells. These recipient bacterial strains were created previously by inserting a 2-lox landing pad (LP) that carries T7RNAP and LacI genes into the recipient genome [103]. The DCB operons were then recombined into the LP through the Cre-lox recombination. Since the LP carries a kanamycin-resistant gene and the pR6K-2L-DCB carries an apramycin resistance gene, we verified the integration of DCB operon into the LP by the gain of the apramycin resistant phenotype and the loss of the kanamycin resistant phenotype of the transformed cells.

### Expression of the DCB genes in the CRAGE system

The CRAGE DCB^+^ strains and their LP counterparts (lacking the DCB insert) from glycerol stocks were inoculated into 40 ml of LB media with 10 µg/ml of apramycin and cultured overnight at 28 °C. The next day, 2 ml of each strain was combined with 38 ml of fresh LB media containing apramycin and cultured to reach OD 600 of about 0.1. One milliliter of each sample was used for DNA extraction by alkaline lysis as described above. Then, the culture tubes with DCB + strains were centrifuged 3900 g for 10 min, the supernatants were discarded, and the pellet was washed with M9 medium and centrifuged again. The pellets were resuspended in 40 ml of M9 media and incubated at 28 °C for 3 h. Afterwards, 0.01 mM of IPTG was added and the cultures were incubated for 3 days. The centrifuged pellet was used for the RNA extraction using the RNeasy kit (74,104, Qiagen).

The SuperScript™ IV First-Strand Synthesis System (18,091,050, Invitrogen) was used for removal of residual DNA and synthesis of the cDNA with random hexamers. The reaction was purified with PCR clean-up magnetic beads (UC Berkeley DNA sequencing facility). The initial qPCR quantification on LightCycler 480 (Roche) aimed to find the largest difference of cycle thresholds (∆Ct) between the copies of the *dcbD* gene in cDNA compared to genomic DNA from each of the CRAGE DCB^+^ strains: 1 ng/µl of cDNA or DNA was used in 12.5 μl reactions of KAPA SYBR Fast kit (KK4611, KAPA) using the following amplification program: 95ºC 5 min, 38 cycles of: 95ºC 30 s, 57ºC for 30 s, and 72ºC for 30 s, followed by 72ºC for 5 min and the melting curve analysis. The primers were suitable for amplification of the *dcbD* gene of the CRAGE strains as well as *Ca.* D. californiensis: univDcbD-F: 5′-ACG CTA AAA TGA CTT ACA TTC CC-3′ and univDcbD-R: 5′-AAT ATA CTT GGC GTT CTT TCC AC-3′. The DNA extracted from the LP strains lacking the *dcbD* was used as negative controls. *Aeromonas piscicola* had the largest ∆Ct and was thus used for the following experiment.

*A. piscicola* DCB^+^ and *A. piscicola* LP were cultured overnight at 28 °C as described above, and the next day the whole volume was transferred into 1 l of LB with apramycin. After 3 h, the culture was centrifuged twice to remove the LB medium and replace it with M9, when a small aliquot of the pellet was taken and stored in − 20ºC for subsequent RNA extraction. The culture was split into four flasks with 500 ml M9 medium each, and after 3 h culture at 28 °C, four different concentrations of IPTG were added to each flask: 0, 0.01, 0.1, and 1 mM (in a follow-up experiment 0.01, 0.001, and 0.0001 mM IPTG concentrations were tested). After 3 days, the cultures were centrifuged, and the RNA was extracted as described above. The qPCR was targeting expression of all six genes in the DCB cluster; these expression values were compared to expression of *A. piscicola*-specific housekeeping genes *IhfA* and *IhfB*, using the same qPCR system as described above, with different amplification conditions: 95ºC 5 min, 38 cycles of: 95ºC 30 s, 65ºC for 30 s, and 72ºC for 30 s, followed by 72ºC for 5 min and the melting curve analysis. Dilutions of the DNA extracted from *A. piscicola* DCB^+^ served as a positive control standard curve.

Finally, 3-l cultures (in two flasks, 1.5 l each) of *A. piscicola* DCB^+^ and *A. piscicola* LP were prepared for quantification of the DCB gene expression after 3 h (before adding IPTG) and 3 days of incubation with 0.01 mM IPTG, using the same qPCR conditions as for IPTG concentration testing. These cultures were also analyzed by LCMS and FACS, as described below.

### Detection of DCB expression in *D. fulva* nudibranch

Frozen excisions from the mantle and digestive tract of Df07 were disrupted with a tissue homogenizer. RNA from 100 μl of cell suspension was extracted using the QuickRNA Fungal/Bacterial Microprep Kit (R2010, Zymo Research), with the following modifications: the skin samples were resuspended in 800 μl RNA Lysis Buffer and the mixture transferred into a ZR BashingBead Lysis Tube. Samples were processed with a Biospec Mini-Beadbeater (Biospec, Bartlesville, OK), a high-speed homogenizer/cell disrupter, for 1 min at the “homogenize” setting. The samples were then centrifuged for 1 min at 13,000 × g to pellet debris, and 600 μl of lysate was transferred into a Zymo-Spin IIICG Column in a collection tube. The manufacturer’s protocol was followed thereafter. SuperScript™ IV First-Strand Synthesis System (18,091,050, Invitrogen) was used for removal of DNA and synthesis of cDNA using random hexamers as described above. The qPCR was performed using the DNA from *A. piscicola* DCB^+^ strain as a positive control for the *dcbD* gene. The expression of the *dcbD* gene using the same qPCR conditions described above, except that the *dcbD* expression was compared with *Ca. D. californiensis*-specific *ihfB* gene amplified with primers Dc-IhfB-F: 5′-CGG CTG AAG TTG TCA GCGA-3′ and Dc-IhfB-R: 5′-ACC ACG CTG ATT GGC TTT TG-3′.

### Extraction and LC–MS/MS of non-polar metabolites

Five-hundred ml of each culture, *A. piscicola* DCB^+^ and *A. piscicola* LP, was lyophilized, dissolved in methanol, and filtered to remove insoluble material, then dried. To remove remaining salts, the samples were dissolved in 5 ml water and applied to an Agilent Bond Elut C18 10 g SPE column, and washed with 3 column volumes of water, then all metabolites were eluted with 100% methanol. These samples were dried in SAVANT SPD111 SpeedVac concentrator (Thermo Scientific), dissolved in 1 ml MeOH, then analyzed by LCMS. A 2-µl aliquot of each sample was injected into an Agilent Zorbax Eclise Plus C18 column (2.1 × 50 mm) at 60 °C with a flow rate of 0.4 ml/min. The gradient run started with 100% buffer A (100% water with 0.1% formic acid) for 1 min, then increased to 100% buffer B (acetonitrile with 0.1% formic acid) over 7 min and held at 100% B for 1.5 min. LCMS data were collected using a Thermo Orbitrap IDX Tribrid (Thermo Scientific) mass spectrometer in centroid format for both positive and negative ion modes with a MS range from 80 to 1200 m*/z* at 60,000 resolution. Mass spectrometer source settings included a sheath gas flow rate of 50 (au), auxiliary gas flow of 10 (au), sweep gas flow of 1 (au), spray voltage of 3.5 kV for positive and 2.5 kV for negative, and capillary temperature of 350 °C.

A molecular network was created with the Feature-Based Molecular Networking (FBMN) workflow [104] on GNPS [105]. The mass spectrometry data were first processed with MZMINE2 v2.53 [106], and the results were exported to GNPS for FBMN analysis. The data were filtered by removing all MS/MS fragment ions within ± 17 Da of the precursor m/z. MS/MS spectra were window filtered by choosing only the top 6 fragment ions in the ± 50 Da window throughout the spectrum. The precursor ion mass tolerance was set to 0.01 Da and the MS/MS fragment ion tolerance to 0.02 Da. A molecular network was then created where edges were filtered to have a cosine score above 0.70 and more than 3 matched peaks. Further, edges between two nodes were kept in the network if each of the nodes appeared in each other’s respective top 10 most similar nodes. Finally, the maximum size of a molecular family was set to be unlimited, and the lowest scoring edges were removed from molecular families until the molecular family size was below this threshold. The spectra in the network were then searched against GNPS spectral libraries [105, 107]. The library spectra were filtered in the same manner as the input data. All matches kept between network spectra and library spectra were required to have a score above 0.4 and at least 3 matched peaks. The molecular networks were visualized using Cytoscape v3.7 [108].

### Evidence for KC-12 affinity to the DCB core biosynthetic genes

The *A. piscicola* DCB^+^ and wild type strain were cultured for 3 days as described above. Ten milliliters of aliquots of cultures were labeled either with 1 μl of 0.1 M KC-12 probe or 1 μl of 0.1 M NC probe. Two milliliters of formaldehyde at a concentration of 16% was added to each tube after the incubation, and the samples were incubated at 4ºC overnight in the dark. The next day, the formaldehyde was removed by centrifugation at 3900 g for 10 min and replaced by PBS. The cells were analyzed on BD Influx™ system (BD Biosciences, San Jose, USA) used with a 70 μm nozzle, and cells were visualized on bi-plots showing side-scatter vs. 430–470 nm fluorescence.

## Results

FACS analysis of bacterial cell homogenates from *D. fulva* nudibranchs Df01 and Df02 showed that samples of gills, gut, and gonads did not produce remarkable blue fluorescence from KC-12 (Supplementary Fig. S2). However, in the skin sample, approximately 18% of viable cells were fluorescent (5% of total events), which allowed enough events to enable sorting and subsequent whole genome amplification of single-cells and multi-cell sorts (Fig. 1e). Two thirds (65%) of wells reached sufficient DNA amplification, and from this subset, 61% passed quality controls after sequencing. This led to the genome analysis of one 10-cell, two 25-cell, seven 50-cell, and four 100-cell sorts (Supplementary Table S4).

**Fig. 2:**
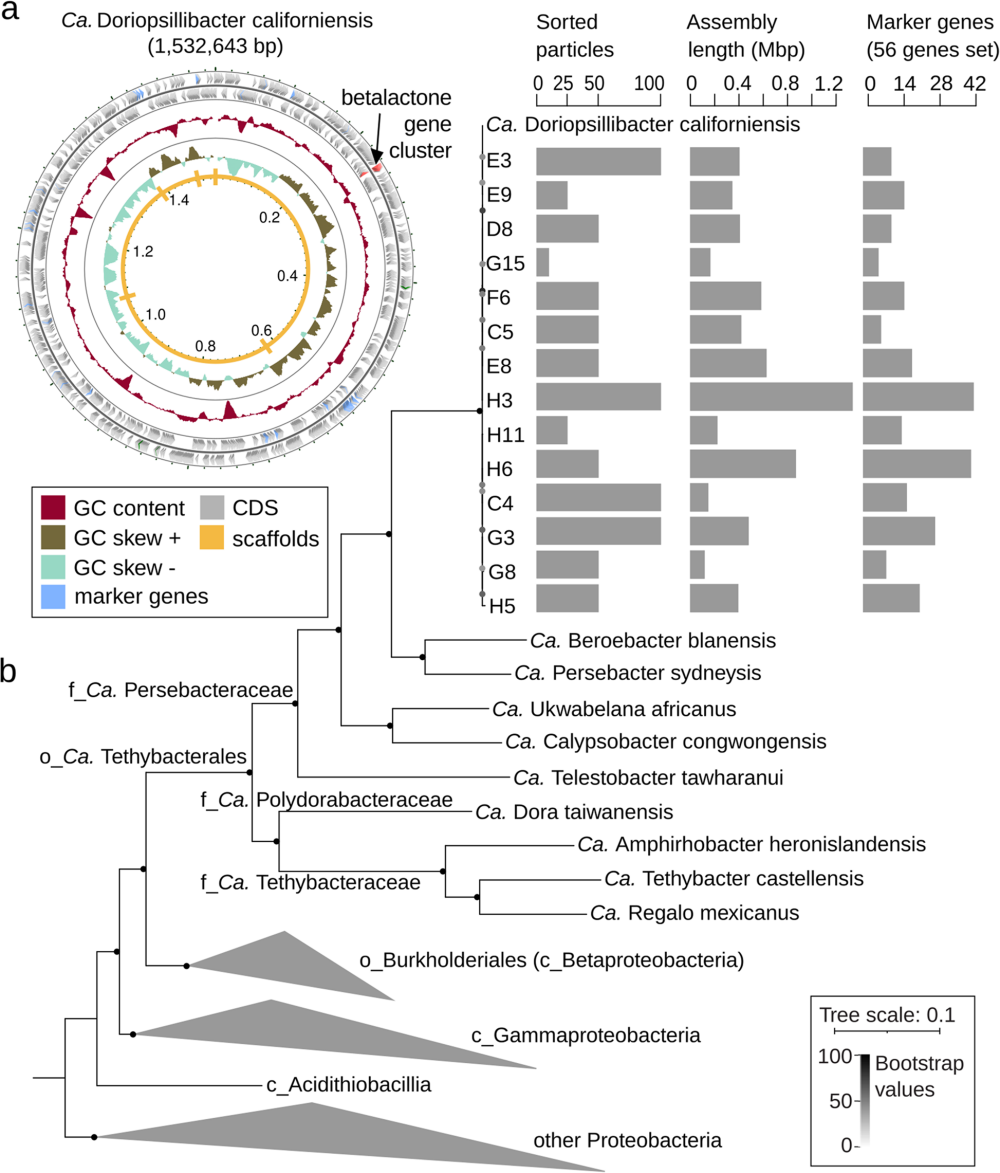
*Ca.* D. californiensis genome. **a** Map of the *Ca.* D. californiensis genome obtained by the co-assembly of sequences from nine cell sorts.** b** Phylogenetic tree based on the 56 marker genes of the co-assembly, individual assemblies of 14 sorts, 9 previously described *Ca.* Tethybacterales medium quality MAGs, and representatives of each family from the Proteobacteria phylum. The number of sorted particles per sort, their genome assembly lengths, and number of detected marker genes are shown

The assemblies of the 14 multi-cell samples ranged from 115 to 1.3 Mbp (median 0.4 Mbp), and the estimated genome completeness averaged 27.9 ± 19.1% with nearly no genome contamination (median contamination 0%, max. 2.4%; Supplementary Table S4). Each sample contained in a single bin, and the unbinned contigs belonged to the mitochondrial DNA of nudibranch. The 100-cell sample H3 had the highest genome completeness (76.5%), encompassing a single 1.3 Mbp bin consisting of 39 contigs. To improve the genome completeness of the bin H3, all reads from other samples that had higher than 99% sequence similarity to H3 on more than 95% of their total assembly length were combined into a co-assembly. The resulting genome assembly was 1,532,643 bp long and contained all 56 single-copy marker genes used for taxonomic classification, distributed along the five final scaffolds (Fig. 2a). Sequences of all 14 samples were very similar to the resulting co-assembled genome: on average 98.2 ± 1.8% of their assembly length matched the final co-assembly with > 99.9% sequence identity, indicating that all sorted cells belonged to the same species (Fig. 2b). We detected only very low strain-level diversity among the sorted cells: 94 SNPs and 26 indels were called across the 14 samples; these variants accounted for up to 0.007% of positions with > 30 × coverage, and 29 of the variants were confirmed in 2–5 samples (Supplementary Table S5).

### First member of the *Ca.* Tethybacterales order from a nudibranch

Phylogenomic analysis based on 56 marker genes showed that the KC-12-enriched species belonged to the recently discovered *Ca.* Tethybacterales, a proposed order of Gammaproteobacteria represented solely by MAGs from sponge microbiomes [38, 39]. Prior to our study, there were 10 genera belonging to three families: *Ca.* Tethybacteraceae (3), *Ca.* Persebacteraceae (5), and *Ca.* Polydorabacteraceae (2). The results of the phylogenetic analysis and average amino acid identity (AAI) of 45–53.5% indicated that the species discovered by our KC-12-guided cell-sorting approach represents a novel genus of the *Ca.* Persebacteraceae family (Fig. 2b). We here propose the name *Candidatus* Doriopsillibacter californiensis acknowledging its host genus and the geographic location of discovery (Supplementary Note 1).

KEGG functional annotation of the *Ca.* D. californiensis genome identified many similarities to metabolic pathways in the other 9 members of the *Ca.* Tethybacterales order with high quality genomes, with few exceptions (Supplementary Fig. S3). Of all members within the *Ca.* Tethybacterales order, *Ca.* D. californiensis encoded the most complete sulfur metabolism: it was predicted to convert sulfite to sulfate, reduce sulfite to sulfide, oxidize sulfide to sulfur, and it also harbored the complete set of the SOX complex genes necessary for the thiosulfate oxidation to sulfate (Supplementary Fig. S4). In contrast, despite its higher genome completeness (99% estimated by CheckM), *Ca.* D. californiensis did not contain genes for nitrate reduction, which were present in other *Ca.* Tethybacterales members with less complete genomes (55–86%). Comparable to other members of the *Ca.* Persebacteraceae family, *Ca.* D. californiensis harbored genes for the transport of various amino acids, phospholipids, heme, iron, spermidine, putrescine, and taurine, while it lacked genes for transporters of mono- and oligosaccharides that were present in the other two *Ca.* Tethybacterales families (Supplementary Fig. S5).

### Low prevalence of *Ca. *D. californiensis sequences across extant datasets

To assess the prevalence and abundance of *Ca.* D. californiensis and its close relatives of across extant public datasets, we mined the Integrated Microbial Genomes and Microbiomes (IMG/M) database, which at the time of analysis contained 18.8 Tbp of sequence data. Interestingly, this IMG/M search did not detect any proteins with best matches to *Ca.* D. californiensis, indicating that its relatives are not abundant enough to be recovered by shotgun metagenomic sequencing.

To further mine for *Ca.* D. californiensis sequences, we next searched for its closest relatives in the Integrated Microbial Next-Generation Sequencing database (IMNGS) containing 422,877 sequencing runs of partial 16S rDNA amplicons from a broad range of environments [73]. In addition, we included sequences, not yet deposited into the IMNGS database, from 41 amplicon runs from 14 nudibranch species from an Indo-Pacific coral reef (described in Cleary et al. [29]) and 17 amplicon runs from 5 nudibranch species from the Red Sea (Abdelrahman et al. [30]). The two studies involved skin and gut samples from eight nudibranch genera from the families Chromodorididae, Discodorididae, and Phyllidiidae*.* We extracted reads that matched *Ca.* D. californiensis with > 92% sequence similarity, which, according to Yarza et al. [74], represents a family-level cutoff of full length 16S rDNA. No exact matches to *Ca.* D. californiensis 16S rDNA were detected. Extracted sequences were then used to construct a 16S rDNA-based phylogeny involving full-length 16S rDNA sequences from *Ca.* Tethybacterales members and other Proteobacteria.

**Fig. 3:**
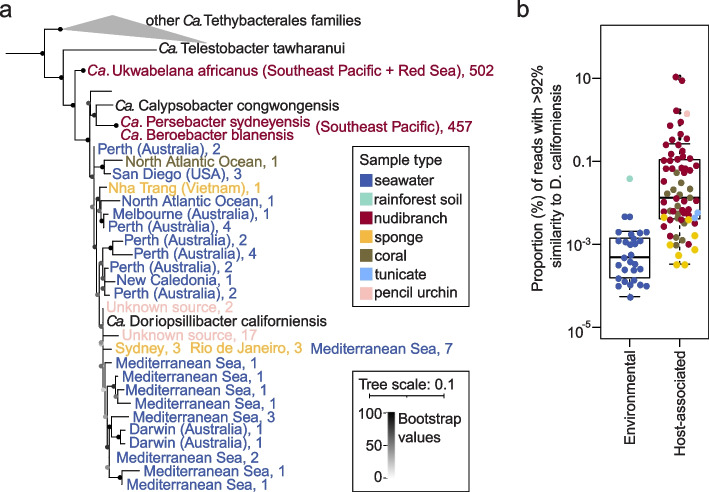
Prevalence of 16S rDNA amplicon sequences with > 92% sequence similarity to *Ca.* D. californiensis in public datasets. **a** Phylogenetic tree of 16S rDNA amplicon sequences from IMNGS and previous nudibranch microbiome studies matching *Ca.* D. californiensis at an approximate family level cutoff and full-length sequences from *Ca.* Tethybacterales. The number of matched reads in each sequencing run is listed after each taxon. The full version of the tree is shown in Supplementary Fig. S6. **b** Proportion of *Ca.* D. californiensis-matched reads in samples from IMNGS and previous nudibranch microbiome studies, visualized on a logarithmic scale for the *y*-axis. The sample types are colored as indicated in the legend

We found that only 56 out of 422,877 samples in the IMNGS database (0.01%) contained reads that mapped to the *Ca.* D. californiensis 16S rDNA sequence; these 56 samples were mostly derived from seawater or soft-bodied marine animals, such as sponges and corals, and as few as 0.00006–1.46% (median 0.004%) of the total reads within the sample datasets matched the query; thus, these reads could have been easily overlooked (Supplementary Table S6). The cluster of the closest relatives of *Ca.* D. californiensis in this 16S rDNA phylogenetic tree comprised 69 partial 16S rDNA sequences from 26 samples of seawater, three sponges (*Hymeniacidon*, *Neofibularia*, and *Scopalina*), one sample associated with the coral *Lophelia pertusa* and one unspecified pencil urchin gut sample (Fig. 3a, Supplementary Fig. S6). However, the absence of full 16S rDNA sequences for these samples prevented the unambiguous assignment of these sequences to the *Ca.* Doriopsillibacter genus. The *Ca.* D. californiensis cluster comprised reads from mostly seawater samples, whereas the reads from the host-associated samples were more similar to other members of the *Ca.* Persebacteraceae family: *Ca.* Ukwabelana africanus and *Ca.* Beroebacter blanensis (Supplementary Fig. S6).

In the nudibranch samples from the two previous studies (Cleary et al. [29] and Abdelrahman et al. [30]), we detected no sequences similar to the *Ca.* D. californiensis cluster but did detect, in 72% of samples that included 8 nudibranch genera, sequences with similarities to *Ca.* Ukwabelana and *Ca.* Beroebacter or *Ca*. Persebacter (Fig. 3a, Supplementary Fig. S6, Supplementary Table S7). However, similar to the IMNGS datasets, the matched reads formed only 0.05% (median) of the total reads in the nudibranch skin or gut microbiomes (Fig. 3b, Supplementary Table S7). The only exceptions were mantle samples from *Goniobranchus annulatu*s and *Chromodoris quadricolor*, in which 9% and 11% of reads were assigned to the *Ca.* Ukwabelana cluster, respectively (Supplementary Fig. S7). This suggests that other members of the *Ca.* Persebacteraceae family are also able to colonize the mantle of nudibranchs. Except for the two *Goniobranchus* and *Chromodoris* skin samples mentioned above, the general proportion of the reads having > 92% sequence similarity to *Ca.* D. californiensis in nudibranch samples and IMNGS amplicon runs was very low. Nevertheless, the host-associated samples contained significantly more matched reads (median 0.01%) than the seawater samples (median 0.0005%, Welch Two sample *t* test, *p* = 0.05; Fig. 3b).

In summary, no reads with 100% sequence similarity with *Ca.* D. californiensis 16S rDNA were found in the microbiome databases and previously sequenced nudibranch microbiomes. *Ca.* D. californiensis’s closest relatives were detected in low abundances in few marine-associated samples and were absent in the nudibranch microbiomes sequenced previously. The inclusion of the reads with family-level similarity to *Ca.* D. californiensis revealed that the nudibranchs sequenced in previous studies contained a low abundance of other members of the *Ca.* Persebacteraceae family.

### Taxonomic composition of the *D. fulva* microbiome and abundance of *Ca*. D. californiensis

**Fig. 4:**
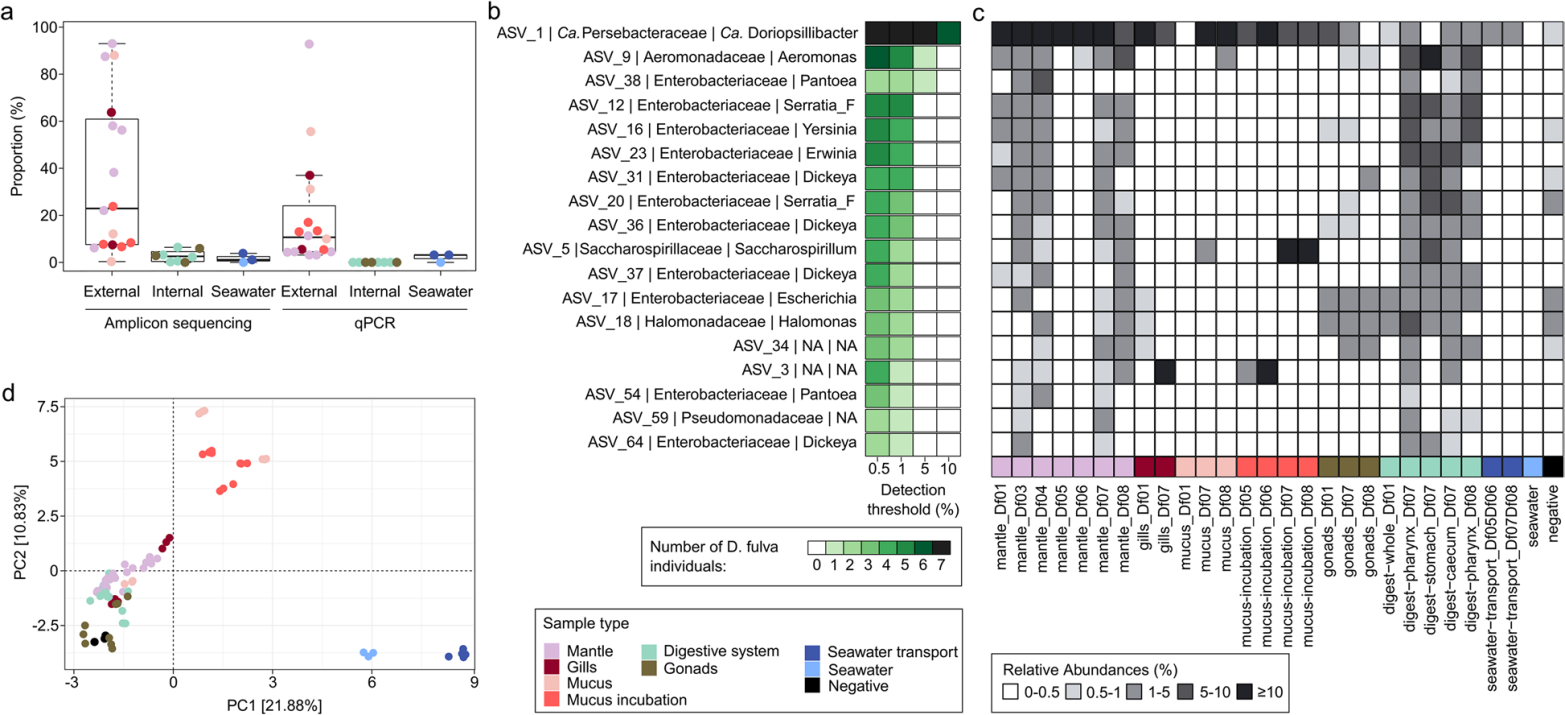
**a** Proportion of *Ca.* D. californiensis reads in microbiomes from different organs of seven *D. fulva* nudibranchs and related water samples estimated by 16S rDNA amplicon sequencing and qPCR. **b** Bacteria (ASVs) of the core mantle microbiome, as defined by different proportion thresholds. The figure shows a subset of the core mantle microbiome ASVs, constituting > 0.1% of the total microbiome. The full version of the core mantle microbiome is shown in Supplementary Fig. S9. Shades of green indicate the number of *D. fulva* specimens having a proportion of the listed ASV above the given threshold. **c** Heatmap visualizing the relative abundances of the ASVs from *D. fulva* core mantle microbiome shown in panel **b** in different samples from the seven *D. fulva* specimens and associated water samples. **d** PCA plot showing ordination of all samples, details are in Supplementary Fig. S10

Next, we explored the overall microbiome diversity and the abundance of *Ca.* D. californiensis in different body parts of *D. fulva* and its surrounding seawater using the following approaches: Sanger sequencing of full-length 16S rDNA amplicon clones, Illumina sequencing of V3 and V4 regions of the 16S rDNA, and qPCR and FISH using *Ca.* D. californiensis-specific probes. We collected 6 additional *D. fulva* specimens from the same Pillar Point sampling site (Supplementary Table S8) and verified specimen identities by sequencing marker genes. Illumina 16S rDNA amplicon sequencing revealed that *Ca.* D. californiensis made up 51.6 ± 32.1% of the total mantle microbiome; its high abundance in all mantle samples was confirmed by qPCR (Fig. 4a). The percent read abundance of *Ca.* D. californiensis ranged between 0.3 and 88% (average 24%) in all *D. fulva* body surface samples: gills, mucus covering the mantle, and in the mucus produced in the laboratory during overnight incubation with KC-12 (Fig. 4, Supplementary Fig. S8). The gills and mucus contained similar or lower proportions of *Ca.* D. californiensis than the mantle samples of the same nudibranchs (29 × lower on average), except for the specimen Df08, which had 14 × more *Ca.* D. californiensis in mucus compared to its mantle. These data suggest that the main reservoir *Ca.* D. californiensis is the nudibranch mantle and that bacterial cells might be released to the surrounding water by the production of mucus. We found *Ca.* D. californiensis at an abundance of 2.8 ± 1.2% of reads in the seawater (200 ml) used to transport the nudibranch from the sampling site to the laboratory (confirmed by both amplicon sequencing and qPCR, Fig. 4, Supplementary Fig. S8). Samples from the nudibranch digestive system and gonads contained only traces of *Ca.* D. californiensis (Fig. 4, Supplementary Fig. S8), which suggests that the food chain or reproduction are likely not the principal means of *Ca.* D. californiensis transmission.

*Ca.* D. californiensis was the only species comprising > 5% of the total mantle microbiome in all sequenced *D. fulva* specimens (Fig. 4b, Supplementary Fig. S9). Apart from *Ca.* D. californiensis, the mantle microbiome composition was very diverse: from a total of 1091 amplicon sequence variants (ASVs) detected in the *D. fulva* mantle, only 28 ASVs had a proportion higher than 0.01% in each of the 7 host specimens (Supplementary Fig. S9). This “core mantle microbiome” was composed of *Ca.* D. californiensis and ASVs of other Gammaproteobacteria, such as *Aeromonas*, *Pantoea, Serratia_F,* and two unclassified ASVs (Fig. 4b, Supplementary Fig. S9). The core ASVs of the mantle that belonged to genera other than *Ca.* Doriopsillibacter were found in higher proportion in nudibranch internal organs (28.4 ± 21.7%) as compared to the mantle (18.9 ± 14.4%, Fig. 4c, Supplementary Fig. S9).

The seawater samples (the seawater in which nudibranchs were transported and the control seawater sample) showed clear compositional differences when compared to samples from nudibranch organs. In the principal component analysis (PCA), 21.88% of the variance was explained by PC1, which was characterized by high abundance of 29 marine ASVs in the seawater samples, such as *Amylibacter*, *Planktomarina*, *Psychromonas*, *Thioglobus*, and *Vibrio* (Fig. 4d, Supplementary Fig. S10). These species comprised up to 31.0 ± 11.1% of the seawater diversity, but as few as 3.3 ± 2.2% in nudibranch mucus and 0.2% in nudibranch body organs (Supplementary Fig. S10). The mucus differed from the rest of the samples (PC2) due to high abundances of 33 ASVs belonging to genera *Colwellia*, *Marinomonas*, *Oleispira*, *Shewanella*, and *Vibrio* (Supplementary Fig. S10), all of which were recovered by Sanger sequencing (Supplementary Fig. S11).

### Localization and confirmation of the biosynthetic activity of *Ca.* D. californiensis

**Fig. 5:**
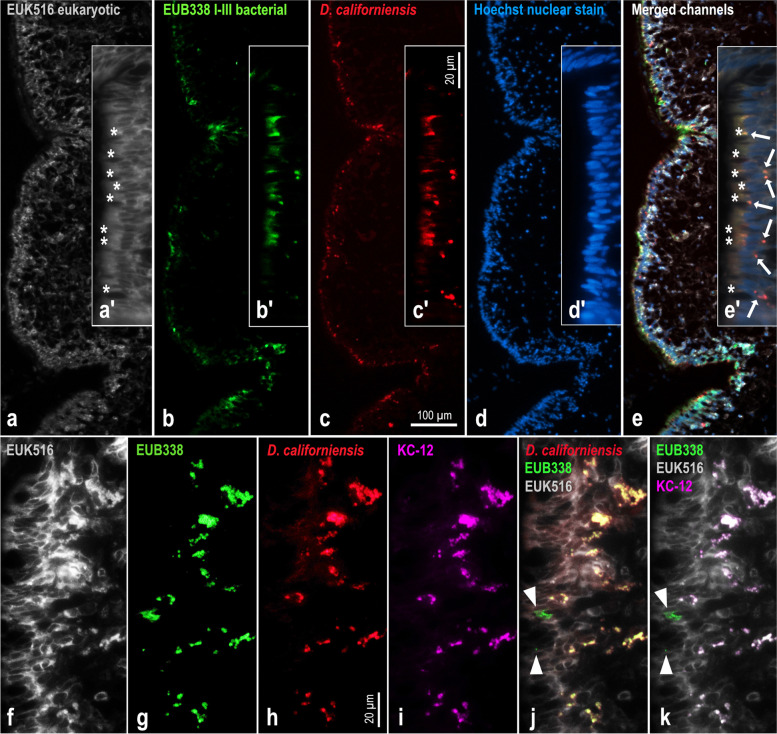
Fluorescent in situ hybridization of *Ca.* D. californiensis in skin tissue of the nudibranch *Doriopsilla fulva*. The upper series of images shows an identical histological section labeled with **a**, **a'** universal eukaryotic probe EUK516 (pseudocolored in gray); **b**, **b'** universal bacterial EUB338 I-III 16S rRNA probe mixture (green); **c**, **c'** *Ca.* D. californiensis-specific 16S rRNA probes (red); and counterstained with Hoechst 33,342 DNA stain (**d**, **d'**; blue). All channels (**a**-**d**, **a'-d'**) are merged in **e**, **e'** showing proportions of *Ca.* D. californiensis (yellow–red) to other bacteria (green). Inserts (**a'-e'**) show localization of *Ca.* D. californiensis within epithelial tissue. *Ca.* D. californiensis (arrows, **e'**) is mostly affiliated with mucus secreting goblet cells (identifiable by large vacuolate space labeled with asterisks: **a'**, **e'**). The bottom series of images shows co-localization of *Ca.* D. californiensis and KC-12 probe signals (**f**–**k**). Eukaryotic EUK516 probe (**f**), bacterial EUB338 I-III probes (**g**), *Ca.* D. californiensis specific probes (**e**), and KC-12 probe (**i**, pseudocolored in purple) are merged in **j** ( *Ca.* D. californiensis, bacterial, and eukaryotic probes) and **k** (bacterial, eukaryotic, and KC-12 probe). Note that bacteria not hybridized with *Ca.* D. californiensis specific probes (arrowheads) also lack the KC-12 probe signal (**j**, **k**)

To confirm the tissue specificity of *Ca.* D. californiensis and to determine its localization within all colonized tissues, we performed fluorescent in situ hybridization (FISH) on histological sections from four specimens of *D*. *fulva* (Df05-08). The signal of *Ca.* D. californiensis-specific 16S rRNA FISH probes revealed that *Ca.* D. californiensis was almost exclusively located in mantle tissues in a rather patchy distribution restricted to the epithelial layer (Fig. 5). Mucus-producing goblet cells (Fig. 5a') appeared to be the primary reservoir of *Ca.* D. californiensis. While clusters of *Ca.* D. californiensis cells were most noticeable at the basal part of goblet cells, a lower cell count was also present at the apical part of epithelial cells (exposed to the external environment) (Fig. 5 c', e'). We could not unambiguously determine whether observed cells of *Ca.* D. californiensis at the apical part of goblet cells were localized intracellularly or on outer cell surfaces. Approximately 70% of the bacterial cells in nudibranch skin emitting green fluorescence of the 16S rRNA universal bacterial probe were hybridized with the far-red-fluorescent 16S rRNA probe specific to *Ca.* D. californiensis (Fig. 5e), which is in accordance with the abundance of *Ca.* D. californiensis obtained by qPCR and 16S rDNA amplicon sequencing (Fig. 4).

The targeted cell sorting assay performed on the first collected nudibranch Df01 indicated that *Ca.* D. californiensis was the only species that integrated the KC-12 probe. To confirm this result, we next used imaging to co-localize the KC-12 probe signal with the rRNA FISH signal in situ. Nudibranch specimens Df07 and Df08 were incubated with the KC-12 and NC probe, respectively, as described for the FACS analysis of Df01 (Fig. 1d, Supplementary Table S1). The specificity of metabolite production was analyzed by *Ca.* D. californiensis-specific 16S rRNA FISH probes on the histological sections (details in Methods). The far-red-fluorescent signal of 16S rRNA probe specific to *Ca.* D. californiensis was overlapped by the blue signal of KC-12 (Fig. 5k). Importantly, no other bacterial species in nudibranch skin was labeled with KC-12, which confirmed the specific uptake of the probe by *Ca.* D. californiensis (Fig. 5j, k).

### Discovery of a unique beta-lactone biosynthetic gene cluster

**Fig. 6:**
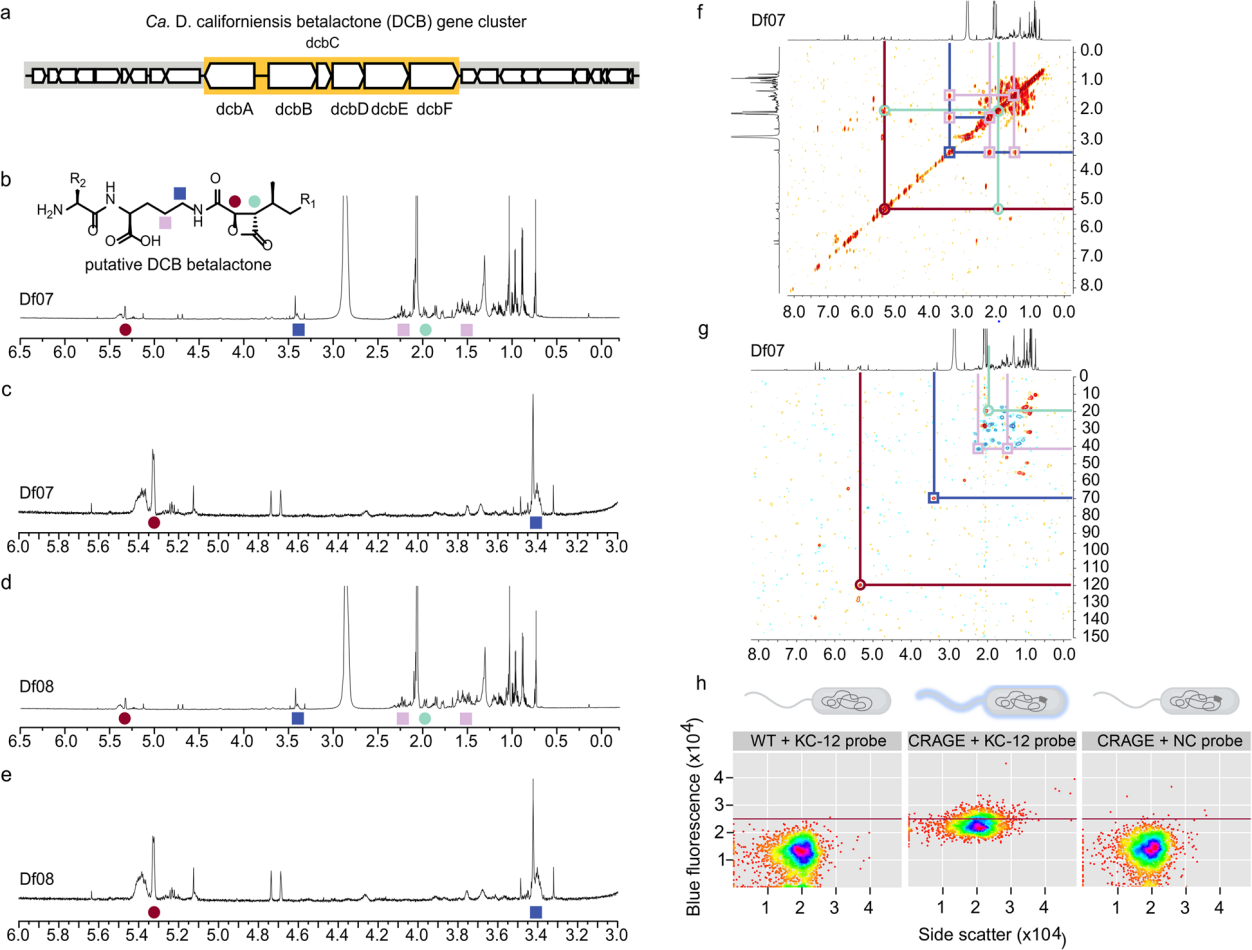
*Ca.* D. californiensis beta-lactone biosynthesis. **a** Schematic of the DCB gene cluster. **b**–**e** Metabolite profiling studies on *D. fulva* skin. NMR spectrum and expansion of the NMR spectrum in CD_3_OD collected from the acetone extract of Df07 and Df08. Note the minor differences in these two spectra. A structure of the putative DCB product is provided along with peak assignments by colored circles or squares. **f** A ^1^H,^1^H-COSY spectrum of the acetone extract of Df07. Correlations between the peaks (colored circles) supported the assignment of each of the four peaks. **g** A ^1^H,^13^C-HSQC spectrum of the acetone extract Df07 skin. Correlations identify the ^13^C chemical shift for the four identified peaks allowing assignment of a putative structure for DCB. **h** Flow cytometry bi-plot of recombinant *Aeromonas piscicola* with CRAGE-inserted DCB cluster labeled with the KC-12 probe and the two negative controls: the same strain stained with the NC probe and the wild type stained with KC-12 probe. Only the combination of the CRAGE strain incubated with the KC-12 probe produced fluorescence (2nd plot). Flow cytometry plots axes are on log scale, *x*-axis shows side scatter signal and *y*-axis blue fluorescence of the probes

The co-assembled *Ca.* D. californiensis genome recovered by the KC-12-targeted cell sorting contained a 27.9-kbp long beta-lactone gene cluster (Fig. 6a). The same cluster was also detected in 11 out of 14 separate assemblies of the multi-cell sorts (Supplementary Table S4), and no SNPs or indels were detected in the cluster’s sequences (Supplementary Table S5). The *Ca.* D. californiensis beta-lactone (DCB) gene cluster was 27.9 kbp and consisted of 25 genes (Fig. 6A, Supplementary File S1).

AntiSMASH (v5.0.0 and v6.1.0), a computational tool for biosynthetic gene cluster detection [100], used with the *Ca.* D. californiensis genome sequence and bacterial reference genome sequences, indicated the DCB sequence was highly unique and identified a set of 17 similar BGCs in reference genomes that included beta-lactones, NRPS and NRPS-like clusters, Type 1 PKS, and hserlactones. However, these BGCs contained only two out of the three core biosynthetic genes of DCB: either a combination of *dcbA* and *dcbF*, or *dcbE* and *dcbF* (Supplementary Fig. S12a). Although other DCB genes were detected in the whole genome sequences, they were located in a distance of thousands of nucleotides from the BGCs, suggesting that they are unlikely to function in these biosynthetic pathways (Supplementary Fig. S12b). The absence of the core biosynthetic genes and low AA sequence similarity of the predicted proteins (average 42%, max 61%) indicates that DCB differs considerably from beta-lactones found in bacterial reference genomes.

To extend our search to the draft bacterial genomes and metagenomes, we performed a blastx search of the 25 individual DCB genes in the NCBI “nr” database. We found proteins with up to 74% AA sequence similarity (average 44%) in 17 different Proteobacteria. The genes for these proteins, however, were not organized in any beta-lactone-like gene cluster in these genomes (Supplementary Fig. S13). Likewise, none of the other nine high quality *Ca.* Tethybacterales genomes contained a BGC similar to DCB (Supplementary Fig. S14); although they contained matches to up to 18 out of the 25 DCB genes (average AA sequence similarity 51%, max 76%), these matches were distributed across the genomes. AntiSMASH detected five different beta-lactones and two other types of BGCs in the *Ca.* Tethybacterales genomes, but they did not share any similarity with the DCB (Supplementary Fig. S14). In summary, *Ca.* D. californiensis contains a beta-lactone gene cluster, which has very few similarities to other previously known beta-lactone-encoding gene clusters and is not found in other members of the *Ca.* Tethybacterales order.

Beta-lactones (structures of 5 examples are provided in Supplementary Fig. S15a) belong to a diverse class of secondary metabolites of high therapeutic value. Little is known about their biosynthetic origin [109], though some similarities with NRPS have been suggested [110]. The biosynthesis of beta-lactones has been described in detail for betalactosine C from *Streptomyces sp.* and cystargolide B from *Kitasatospora cystarginea* [111, 112]. As shown in Supplementary Fig. S15b-c, we were able to identify low AA sequence similarity matches of *dcbA* to the first enzyme in the cystargolide (*cysA*) and belactosin (*belJ*) pathways (29% and 28% AA sequence similarity on 60% and 57% sequence length for *cysA* and *belJ*, respectively). This suggests that the DCB pathway also begins with a Claisen-type condensation by an isopropyl malate synthase (IPMS) to form a 2-isopropylmalate. In addition, *dcbF* matched AA sequences of putative AMP-dependent synthases *cysF* and *belH* (22% and 25% AA sequence similarity on 64% and 28% sequence length for *cysF* and *belH*, respectively), which are proposed to conduct the ultimate amide bond coupling between the beta-lactone core and the corresponding dipeptide arm. While incomplete, we were able to use this comparison to suggest a preliminary pathway for the putative DCB (Supplementary Fig. S15d).

### Metabolite profiling of the *D. fulva* nudibranch tissues

Seeking to detect the metabolic products of DCB, we profiled the metabolites from our nudibranch specimens. As our nudibranch sample biomass was very small (50 ± 10 mg of tissue/organism) and even smaller (20 ± 5 mg) for only the mantle, we turned to capillary NMR methods. Using nudibranch Df03 as a model, we determined that sequential extraction with acetone and methanol provided the best mass recovery (Supplementary Fig. S16). We then applied this to the specimens Df07 (Fig. 6b, c) and Df08 (Fig. 6d, e), obtaining about 10 ± 5 µg, an NMR estimate by ^13^C satellite analyses [113] of crude extract in the acetone extract with < 50% of that obtained from the subsequent methanol extraction. Based on this, ^1^H,^1^H-COSY (Fig. 6f) and ^1^H-^13^C-HSQC ASAP spectra (Fig. 6g) were collected from Df07. Unfortunately, the concentration was too low to collect effective ^1^H-^13^C-HMBC or ^1^H-^1^H-NOESY to complete the assignments. That noted, we were able to tentatively assign peaks to the lactone ring (red and green circles, Fig. 6b, e) as well as to the proximal peptide residue (blue and purple squares, Fig. 6b, e). This assignment was conducted both by hand and automatically using MestreNova v12.0; both methods returned the same assignments.

### Beta-lactone expression in heterologous hosts

To further understand the biosynthetic pathway, we explored the recombinant expression of a synthetic DcbA-DcbF gene cluster using the chassis-independent recombinase-assisted genome engineering (CRAGE) system in nine Gammaproteobacteria hosts, a method developed in our laboratory for inserting BGCs directly into the bacterial chromosome [103]. From the seven CRAGE strains capable of reaching stationary phase overnight, *Aeromonas piscicola* showed the highest levels of DCB expression in M9 media supplemented with IPTG (Supplementary Fig. S17a). The CRAGE DCB^+^ strains served also as positive controls for development of a qPCR assay for the detection of one of the genes involved in DCB biosynthesis (i.e., *dcbD*) in *D. fulva* nudibranch body organs. We detected expression of *dcbD* in nudibranch mantle tissues, while its internal organs did not express the gene (Supplementary Fig. S17b), which was in accordance with the results of our analyses of microbial composition in different nudibranch organs based on 16S rDNA (Fig. 4), as well as the flow cytometry data after applying the KC-12 probe (Supplementary Fig. S1).

DCB gene expression in *A. piscicola* DCB^+^ was enhanced by testing different IPTG concentrations in 3-day-old cultures in M9 medium and compared to a 3-h culture of *A. piscicola* DCB^+^, in which no DCB expression was expected. A 3-day culture of *A. piscicola* lacking the DCB insert was used as a control. All six DCB genes were expressed more efficiently with 0.01 mM IPTG than at higher concentrations (similarly to our previous CRAGE study Wang et al. [103]), reaching 177 × higher expression than *A. piscicola* housekeeping genes (Supplementary Fig. S18). Some endogenous metabolites appeared to be produced in different quantities when comparing supernatant and cell pellet extracts of the 3-day *A. piscicola* DCB^+^ culture and the two negative controls by LCMS and feature-based molecular networking (Supplementary Fig. S19). However, no beta-lactones were identified.

Finally, we used *A. piscicola* DCB^+^ to further support our result that our fluorescent pantetheine analog probe KC-12 has affinity to the products of DCB core biosynthetic genes. Aliquots from the cultures, used for qPCR analysis described above, were labeled with KC-12 or the NC control. As a result, we observed blue fluorescence in the *A. piscicola* DCB^+^ 3-day culture, but not in the negative controls, which demonstrates the utility of the KC-12 probe for detecting active DCB expression (Fig. 6h). Overall, these experiments demonstrate the use of the CRAGE system not only as a tool for metabolite production as shown in our previous study [103], but also and most critically, as a tool to validate the activity of discrete enzymes within a biosynthetic pathway.

## Discussion

Current estimates suggest that < 5% of the known nudibranch species have been studied for their metabolites and < 0.5% for their microbiome composition [24]^.^ Previous 16S-rDNA amplicon-based studies showed that the nudibranch gut and mantle harbor a large portion of uncharacterized bacterial lineages [28–30]. However, the genomic characterization of these bacteria is lagging, which means that the role of these host-associated lineages remains unknown. The only novel species with a complete genome sequenced prior to our study was isolated from foot epidermis of the nudibranch *Glossodoris cincta*; this bacterium, *Sneathiella glossodoripedis*, belongs to Sneathiellales (Alphaproteobacteria), an order of free-living marine bacteria [114]. Apart from that, culture-based studies repeatedly report the same easily culturable genera, such as *Bacillus*, *Marinomonas*, *Pseudomonas*, *Serratia*, and *Vibrio* [31–34]. Not surprisingly, we cultivated these same species when attempting to isolate *Ca.* D. californiensis on selective media plates (data not shown).

For more than two decades, it has been speculated that symbiotic bacteria could play a role in nudibranchs’ chemical defense. Diverse bacteria have been observed by electron or fluorescent microscopy in bacteriocyte-like compartments in the nudibranchs, including members of the genera *Aeolidia*, *Berghia*, *Coryphella*, *Cuthona*, *Dendrodoris*, *Dendronotus*, *Doto*, *Facelina*, *Flabellina*, *Janolus*, *Polycera*, *Rostanga*, and *Tritonia* [26–28, 115, 116]; however, the role of these bacteria in the nudibranch chemical defense system has not been demonstrated. The previous studies involved testing bioactivity of crude extracts from bacterial isolates, though none of these bacterial metabolites have been detected in the direct extracts from the mantle tissues of the same nudibranchs [31–34]. Accumulation of metabolites for chemical defense of bacterial origin has been observed in the nudibranch *Polycera atra*, which feeds on *Bugula neritina,* a bryozoan containing a bacterial symbiont *Endobugula sertula* producing bryostatin; however, the presence of this bacteria in nudibranchs has not been reported [117]. Our study is the first to provide direct evidence that the natural products from bacterial symbionts are detectable in the body of their nudibranch host: NMR analysis of extracts from *D. fulva* mantle supported the presence of a beta-lactone but due to small sample size was not able to complete the subsequent dereplication and structure elucidation process. Ethological studies are necessary to understand how this beta-lactone functions within the context of the *D. fulva* host and its predators.

*Ca.* D. californiensis, the most abundant species in the *D. fulva* mantle, was not detected in any other nudibranchs species from previous studies [28–30], although most species contained sequence traces (less than 0.1%) of other *Ca.* Tethybacterales genera. This is not surprising, since on a global scale we detected only ultra-low amounts of *Ca.* Tethybacterales sequences in data from seawater and marine invertebrates. Taylor et al. [38] and Waterworth et al. [39] recently reported the first *Ca.* Tethybacterales MAGs characterized by heterotrophic features supplying diverse nutrients to their sponge hosts; however, their contribution to sponge chemical defense has not been investigated. From thousands of sponge samples belonging to hundreds of species investigated so far [118], only 27 samples were found to be dominated by *Ca.* Tethybacterales [39]. This highlights the need to further study the prevalence of *Ca.* Tethybacterales across different nudibranch species. It is worth mentioning the variability of the *Ca.* Tethybacterales proportion across nudibranch individuals from the same species; we found a high proportion of *Ca.* Tethybacterales (10%) in only one out of three *Chromodoris* and in one out of four *Goniobranchus* individuals from previous nudibranch studies. Similarly, while all seven *D. fulva* specimens sequenced in our study contained *Ca.* D. californiensis, its proportion in the mantle ranged between 6 and 94%. Further research should explain whether the compositional variability of the nudibranch mantle microbiome is influenced by different mantle sampling approaches, or by environmental factors, such as seasonal variations, nudibranch age, or its mucus production capacity (e.g., Df08 had the highest proportion of *Ca.* D. californiensis in its mucus, but the lowest in its mantle). It has been shown, for example, that the composition of the human skin microbiome changes with increasing sampling depth in the epidermis [119]. If a small piece of nudibranch mantle is sampled as a whole, the marine bacteria from the surface may outnumber true skin symbionts, which could dilute their abundance in the final microbial community profile.

In addition, the transmission of nudibranch symbionts should be investigated in more detail. *Ca.* D. californiensis*-*specific qPCR, FISH, and 16S rDNA amplicon sequencing in this study revealed that the principal reservoir of the *Ca.* D. californiensis is the nudibranch mantle and that this bacterium can be released to the surrounding water via the mucus. The gills filter the surrounding water; thus, they also can contain a considerable proportion of *Ca.* D. californiensis. However, hybridization with the KC-12 probe demonstrated that the active production of secondary metabolites by *Ca.* D. californiensis is restricted to the mantle. *Ca.* D. californiensis is located in mucus-producing goblet cells, similar to the sponge-associated *Ca.* Tethybacterales, which are located in bacteriocytes in sponge mesohyl [38]. This suggests that all *Ca.* Tethybacterales members characterized so far have a host-associated lifestyle, which leads to the question of how they are transmitted from parent to offspring. A recent study on sponges revealed that vertical transmission of sponge-associated microbes is widespread but not universal to all of its bacterial symbionts, with many of them transmitted horizontally [120]. The only previous study focused on transmission routes of symbionts in nudibranchs observed microorganisms in the egg mass of the nudibranch *Dendrodoris nigra*, suggesting maternal transmission of some symbiotic bacteria; however, the role of these microbes in chemical defense has not been clarified [115]. The phylogenetic analysis of the 10 sponge-associated *Ca.* Tethybacterales and their hosts in the study of Waterworth et al. [39] endorses the horizontal community transfer hypothesis for this clade. It seems that the associations between the *Ca.* Tethybacterales members and their sponge hosts originated multiple times over their evolutionary history, because the phylogeny of *Ca.* Tethybacterales is not congruent with the phylogeny of their sponge hosts [39]. The discovery of *Ca.* D. californiensis, the 11th species of the *Ca.* Tethybacterales order, in nudibranchs is not surprising, because sponges either form an important part of the nudibranch diet or live in close proximity to nudibranchs. We captured the release of *Ca.* D. californiensis from mucus-producing goblet cells in a small volume of seawater used for transport and incubation of *D. fulva* in the laboratory. However, the number of *Ca.* D. californiensis cells released to the seawater is negligible compared to all marine bacteria, which makes it nearly undetectable in seawater samples. While our study has shown that *D. fulva* gonads are not the principal localization of *Ca.* D. californiensis, more experiments are needed to confirm the horizontal transmission in the *D. fulva* community.

In the present study, we illustrated the usage of a fluorescent probe of biosynthetic activity for selective isolation and sequencing of bacterial cells from a cellular homogenate. Here, we treated live nudibranchs with the KC-12 probe and, using a KC-12 single-cell genomics strategy, identified *Ca.* D. californiensis as a putative PKS/NRPS active cell. To date, our studies have focused on the use of the KC-12 probe based on its ability to label carrier proteins (CP) associated with fatty acid (ACPs), polyketide (ACPs), and NRPS (PCPs) synthesis [47]. As shown schematically in Fig. 1a, the uptake of KC-12 into a cell can hijack the coenzyme A biosynthetic pathway and be converted to the corresponding CoA-analog. This KC-12 CoA can be post-translationally appended to ACP/PCP through the action of a 4’-phosphopantethinyl transferase generating a fluorescently-labeled ACP/PCP [47]. While no protein in the DCB pathway was predicted to contain an ACP or PCP domain, the putative first step in DCB biosynthesis (Supplementary Fig. S15) involves the condensation of an acyl-CoA, which would likely be inhibited by KC-12 CoA. Using a synthetic biological approach, we successfully demonstrated how the CRAGE system was able to validate this ACP/PCP-independent staining in vivo. Studies are now underway to fully evaluate the use of KC-12 and related ACP/PCP targeting probes in terms of their ability to identify cells with both CP and CP-free (those that use only CoA) pathways.

Finally, and most critically, this study demonstrates a fluorescent probe for confirming in vivo functionality of bacterial BGCs. Computational tools applied to large metagenomic datasets in recent years have resulted in discovery of thousands of novel BGCs, but our ability to confirm their functionality represents the bottleneck for testing their potential use in the pharmaceutical industry or agriculture [121]. While the functionality of BGCs found in culturable bacteria can be examined by analyzing extracts from bacterial cultures, the successful heterologous production of natural products from uncultured bacteria is much more complicated [122]. Such heterologous production requires detailed knowledge on biosynthesis of these secondary metabolites, advanced genome engineering tools and optimization of the expression methods. In addition, lack of metabolites from the hosts can hinder the successful in vitro synthesis of the selected compound [123]. This might be the reason why nearly all natural products isolated from marine host-associated bacteria have come from culturable bacteria [124], and detailed molecular characterization of natural products derived from uncultured microbes is still very rare [10]. Confirmation of the in vivo functionality of BGCs detected in uncultured microbes, demonstrated in the present study by KC-12 probe labeling, can initiate the first step in further biochemical characterization of BGC-encoded products.

## Conclusions

Synthase-selected cell labeling by the KC-12 fluorescent pantetheine probe allowed capturing a new member of *Ca.* Tethybacterales, actively producing secondary metabolites in the mantle of *Doriopsilla fulva* nudibranch*.* This resulted in obtaining the first genome sequence of an uncultured nudibranch symbiont, *Ca.* D. californiensis, which is the first member of *Ca.* Tethybacterales detected in nudibranchs and also the first *Ca.* Tethybacterales genus proven to produce secondary metabolites. It forms part of the core mantle microbiome of *D. fulva* and is transmitted horizontally. Its genome harbors a BGC associated with a beta-lactone. Beta-lactones represent an underexplored group of secondary metabolites with pharmaceutical potential that have not been reported in nudibranchs previously. The same beta-lactone was the only compound detected in *D. fulva* tissues which hints at the possibility of symbiotic microbes playing a role in the chemical defense of this nudibranch species. The present study also illustrated how a fluorescent pantetheine probe implemented in cell sorting can be used to discover secondary metabolites in uncultivated microbial lineages in vivo. While computational analysis of BGCs does not guarantee that the compound is produced in vivo, the synthase-selected cell labeling implemented in this study identifies a previously unknown beta-lactone BGC and unites that discovery with secondary methods to further verify that this pathway is active. Although this study failed to isolate and characterize the structure of ascribed beta-lactone (due to the low sample size), this method offers a unique set of tools to identify associated bacteria and begin to explore their secondary metabolism.

## Supplementary Information

**Additional file 1:** **Supplementary Figure S1.** Photorhabdus luminescens incubated with the KC-12 probe during 70 h. **Supplementary Note 1.** Taxonomic Appendix. **Supplementary Figure S2.** Hybridization of cells from nudibranch skin, gut, gills and gonads. **Supplementary Figure S3.** KEGG modules of Ca. D. californiensis and medium quality Ca. Tethybacterales MAGs from sponges. **Supplementary Figure S4.** Sulfur and nitrogen metabolism of Ca. D. californiensis and medium quality Ca. Tethybacterales MAGs from sponges. **Supplementary Figure S5.** ABC transporters of Ca. D. californiensis and medium quality Ca. Tethybacterales MAGs from sponges. **Supplementary Figure S6.** Phylogenetic tree of 16S rDNA gene sequences from public datasets. **Supplementary Figure S7.** Proportion of reads with >92% sequence similarity to Ca. D. californiensis in other nudibranchs. **Supplementary Figure S8.** Heatmap showing relative abundances of top ASVs across all 16S rDNA amplicon samples. **Supplementary Figure S9.** Core microbiome of D. fulva mantle. **Supplementary Figure S10.** ASVs determining the ordination of samples in the PCA anlaysis of nudibranch microbiome samples. **Supplementary Figure S11.** Phylogenetic tree of full length 16S rDNA gene sequences from nudibranch skin and mucus obtained by Sanger sequencing. **Supplementary Figure S12.** Ca. D. californiensis betalactone gene cluster compared to the best antiSMASH matches. **Supplementary Figure S13.** Similarity of the genes in the Ca. D. californiensis betalactone gene cluster with the 'nr' database of NCBI. **Supplementary Figure S14.** Similarity of the genes in the Ca. D. californiensis betalactone gene cluster with other members of the Ca. Tethybacterales order. **Supplementary Figure S15.** Biosynthetic pathway comparison to known betalactone natural product pathways. **Supplementary Figure S16.** NMR analysis of extracts from D. fulva nudibranchs. **Supplementary Figure S17.** Quantification of the dcbD expression in the CRAGE strains and nudibranch samples. **Supplementary Figure S18.** Testing of the CRAGE strain Aeromonas piscicola for betalactone expression induced by IPTG. **Supplementary Figure S19.** Feature-based molecular network identifying metabolite production in the A. piscicola DCB+ CRAGE strain.

**Additional file 2:** **Supplementary Table S1.** Overview of the collected Doriopsilla fulva nudibranchs and methods applied for their analysis. **Supplementary Table S2.** Synthetic building blocks and PCR primer sequences used for engineering CRAGE strains. **Supplementary Table S3.** Details on CRAGE strains genomes. **Supplementary Table S4.** Assemblies of multi-cell sorts. **Supplementary Table S5.** SNPs and indels in the multi-cell sorts sequence data. **Supplementary Table S6.** 16S rDNA amplicon sequencing runs from IMNGS containing reads with >92% similarity to Ca. D. californiensis. **Supplementary Table S7.** 16S rDNA amplicon sequencing runs from previous nudibranchs microbiome studies containing reads with >92% similarity to Ca. D. californiensis. **Supplementary Table S8.** Collected nudibranch samples and the details on 16S rDNA amplicon Illumina sequencing runs.

**Additional file 3.** Supplementary File S1. DCB antiSMASH output file.

## Data Availability

The sequences and the genome assemblies are accessible through the Integrated Microbial Genomes and Microbiomes website https://img.jgi.doe.gov/ with sequencing project IDs listed in Supplementary Table S4. The genome of *Ca.* D. californiensis is available at NCBI with BioProject ID: PRJNA864331. The Illumina 16S rDNA amplicon sequences have been deposited to EMBL-EBI with BioProject ID: PRJEB55111. The Sanger 16S rDNA reads have been deposited to NCBI with BioProject IDs: PRJNA864631, PRJNA864634, PRJNA864635, and PRJNA864637. The new bacterial taxa name *Doriopsillibacter californiensis* is registered in SeqCode under the permanent link: seqco.de/r:td4s-yi2.
