# Supplementary material for: Synthase-selected sorting approach identifies a beta-lactone synthase in a nudibranch symbiotic bacterium
Source: Microbiome. 2023 Jun 13;11:130. doi: 10.1186/s40168-023-01560-8 (PMC10262491; doi:10.1186/s40168-023-01560-8)

Supplementary Information

Synthase-selected sorting approach identifies a beta-lactone synthase in a nudibranch symbiotic bacterium

Mária Džunková, James J. La Clair, Tomáš Tyml, Devin Doud, Frederik Schulz, Samuel Piquer, Dafne Porcel Sanchis, Andrew Osborn, David Robinson, Katherine B. Louie, Ben P. Bowen, Robert Bowers, Janey Lee, Vicente Arnau, Wladimiro Díaz-Villanueva, Ramunas Stepanauskas, Terry Gosliner, Shailesh V. Date, Trent Northen, Jan-Fang Cheng, Michael D. Burkart, Tanja Woyke

Supplementary Figure S1: ***Photorhabdus luminescens*** **incubated with the KC-12 probe during 70 h.**
The scatterplots show forward scatter vs. blue fluorescence for the samples. The gate is set according to the negative sample at the beginning of the experiment. 10,000 events have been first gated on forward scatter vs. side scatter plot to indicate the area corresponding to bacterial cell removing instrument noise. The numbers (%) show the proportion of the cells in the gated area in the scatterplot.

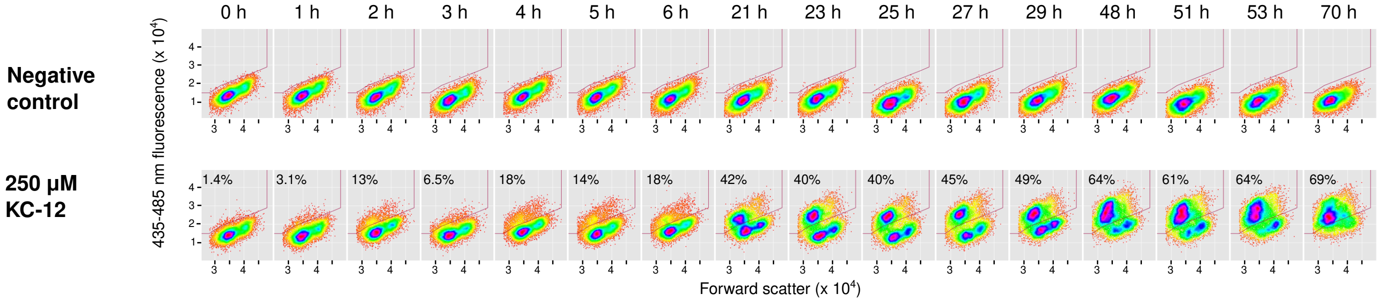

Supplementary Note 1:

Taxonomic Appendix

Description of '*Candidatus* Doriopsillibacter californiensis'. The whole genome-based phylogenies enabled the assignment of the *Doriopsillibacter* genus. We propose to establish a provisional taxon '***Candidatus*** **Doriopsillibacter californiensis**' according to the code of the International Committee on Systematic Bacteriology.

Candidatus **Doriopsillibacter californiensis** (indicating host name and collection location). Belonging to *Candidatus* Persebacteraceae, *Candidatus* Tethybacterales, Gammaproteobacteria. Symbiont of nudibranch *Doriopsilla fulva* (Dendrodorididae, Nudibranchia) collected from a tide pool at Pillar Point, California, USA (37º 29′ 41.427″ N, 122º 29′ 57.994″ W), discovered by synthase-selected single-cell genomics. Mucus-producing goblet cells of *Doriopsilla fulva* appear to be the primary reservoir of *Ca*. D. californiensis. Genome sequence available at NCBI with BioProject ID: PRJNA864331.

Supplementary Figure S2: Hybridization of cells from nudibranch skin, gut, gills and gonads.
The upper scatterplots show the side scatter on the x-axis vs. red nucleic acid stain SYTO 61 on y-axis. Gated events were then visualized on scatterplots showing side scatter on x-axis and blue fluorescence corresponding to the probe KC-12 probe on y-axis.

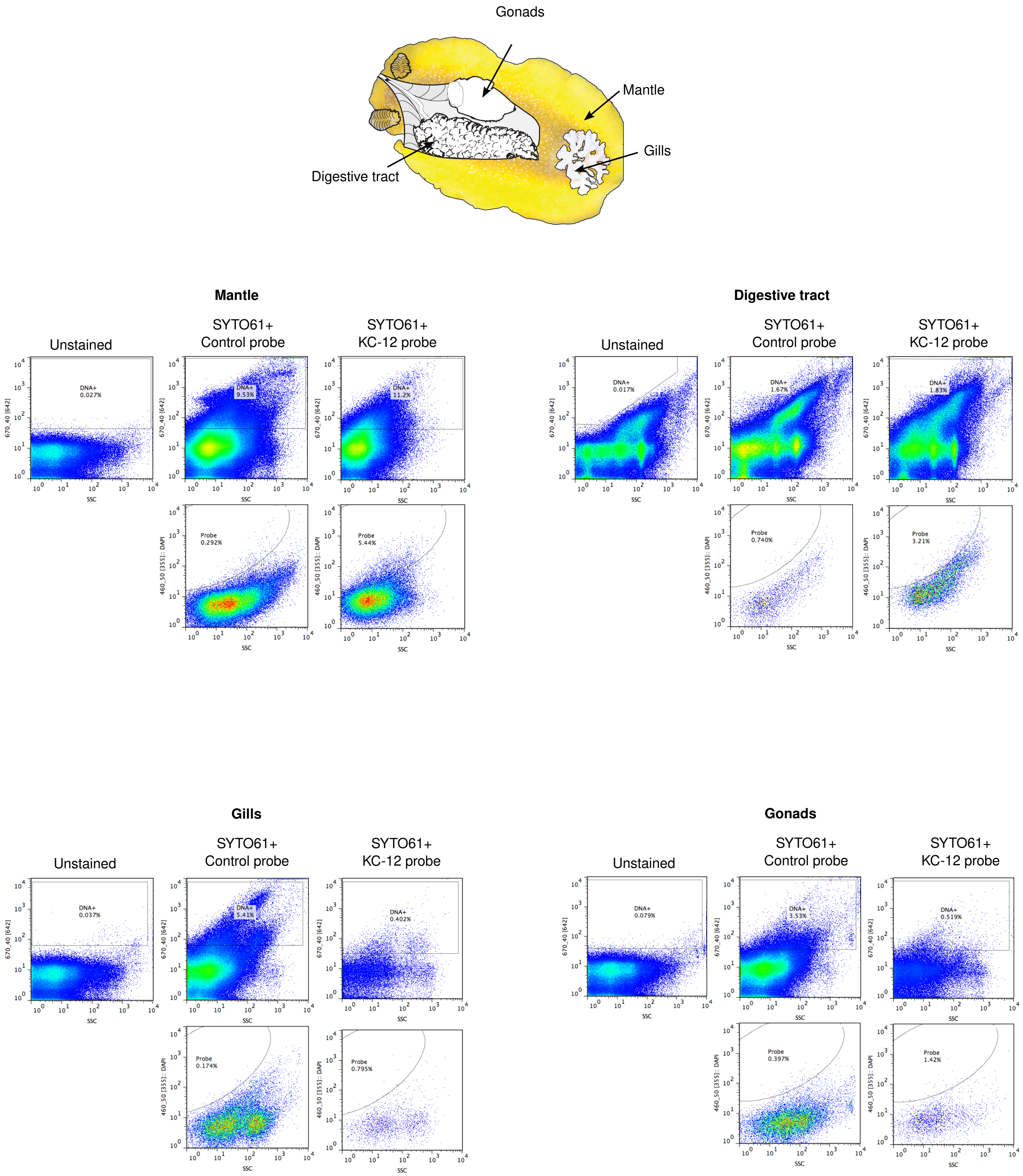

Supplementary Figure S3: KEGG modules of *Ca.* D. californiensis and medium quality *Ca.* Tethybacterales MAGs from sponges. Only the modules that were complete in at least one genome are shown. The phylogenetic tree is based on the whole genome sequences (as shown in the Figure 2).

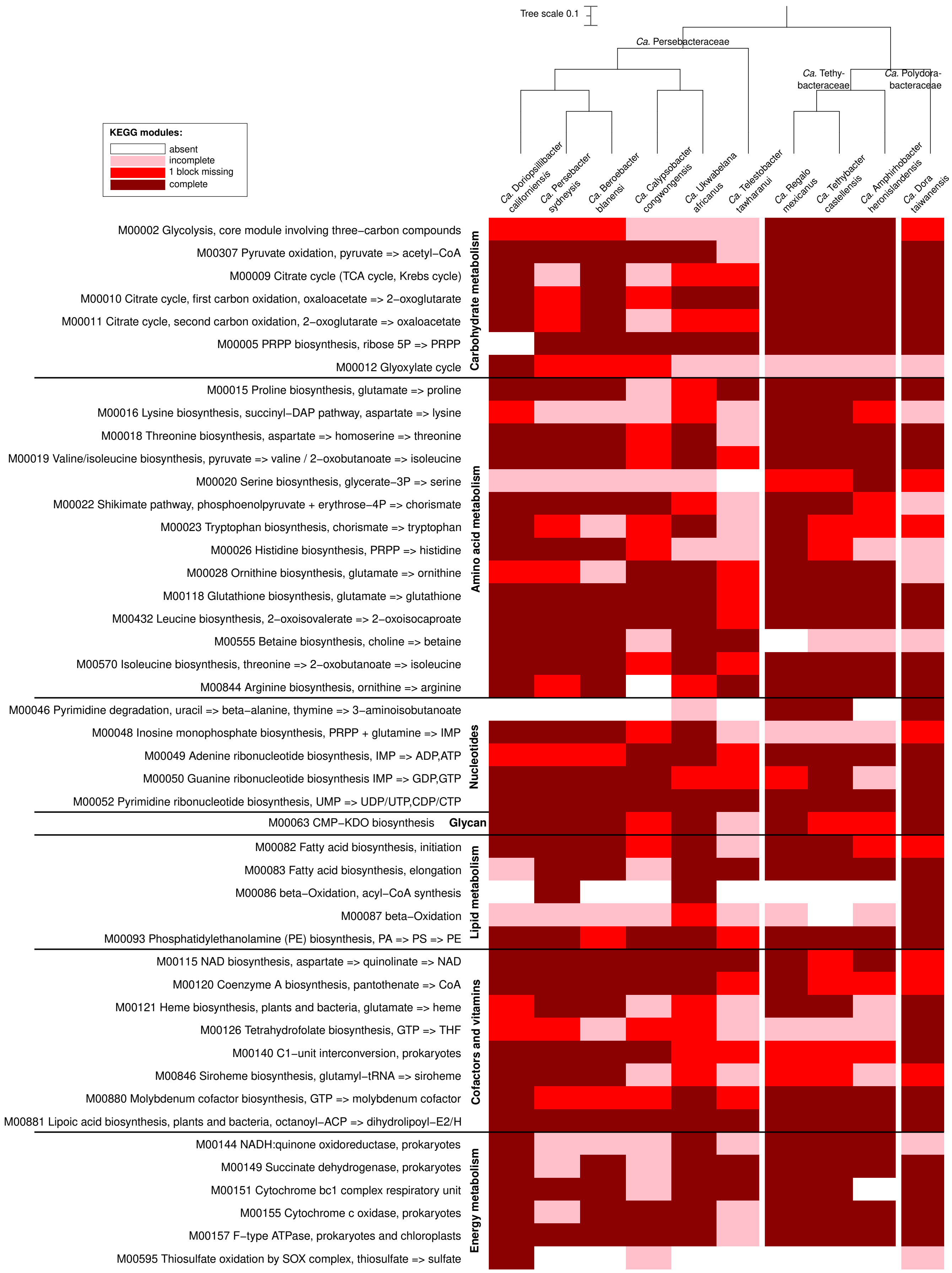

Supplementary Figure S4: Sulfur and nitrogen metabolism of *Ca.* D. californiensis and medium quality *Ca.* Tethybacterales MAGs from sponges.
The colored squares represent genes in the Ca. Tethybacterales genomes matching the KEGG nitrogen and sulfur pathways map.

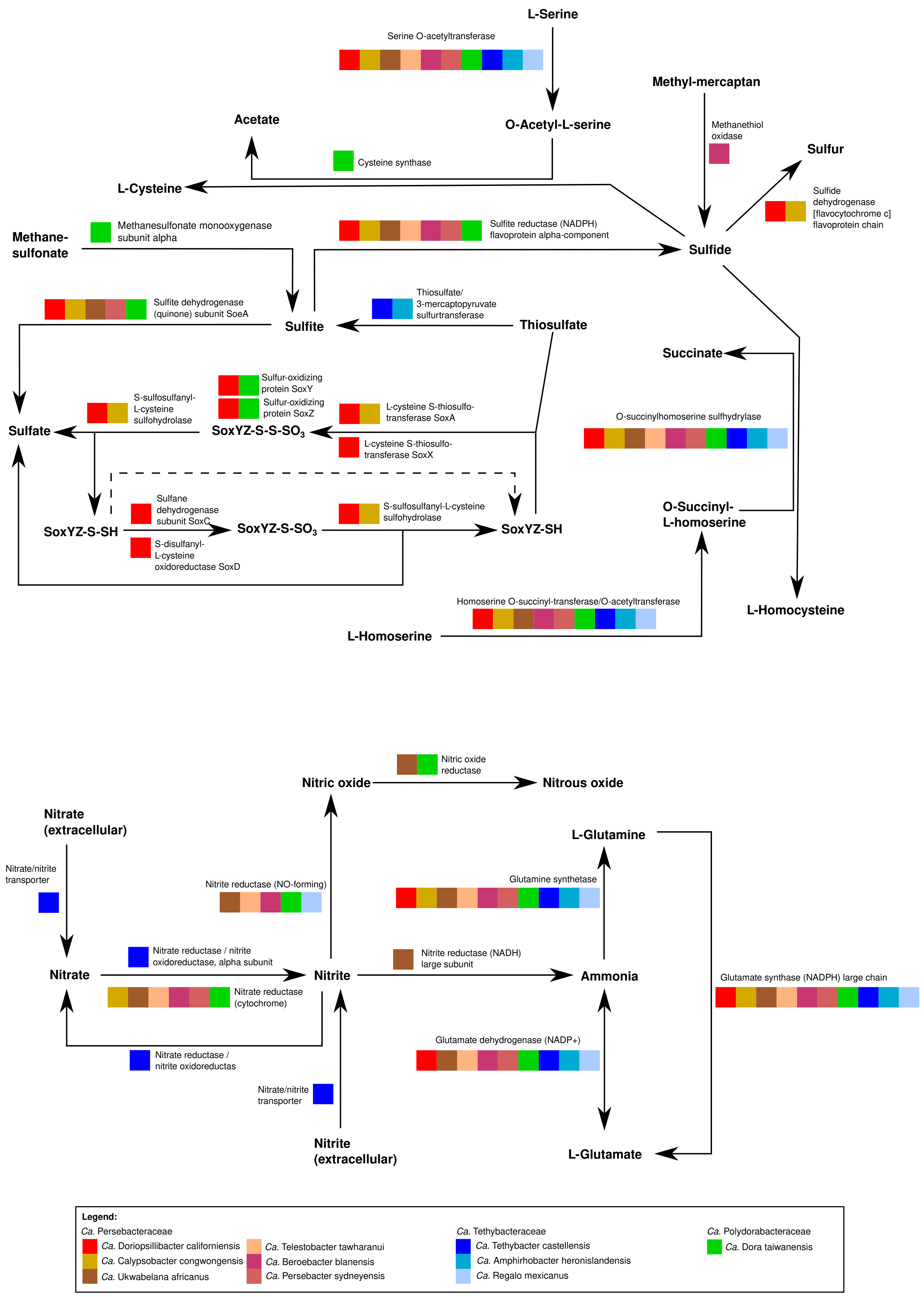

Supplementary Figure S5: ABC transporters of *Ca.* D. californiensis and medium quality *Ca.* Tethybacterales MAGs from sponges. The colored squares represent genes in the *Ca.* Tethybacterales genomes matching the KEGG ABC transporter pathway map.

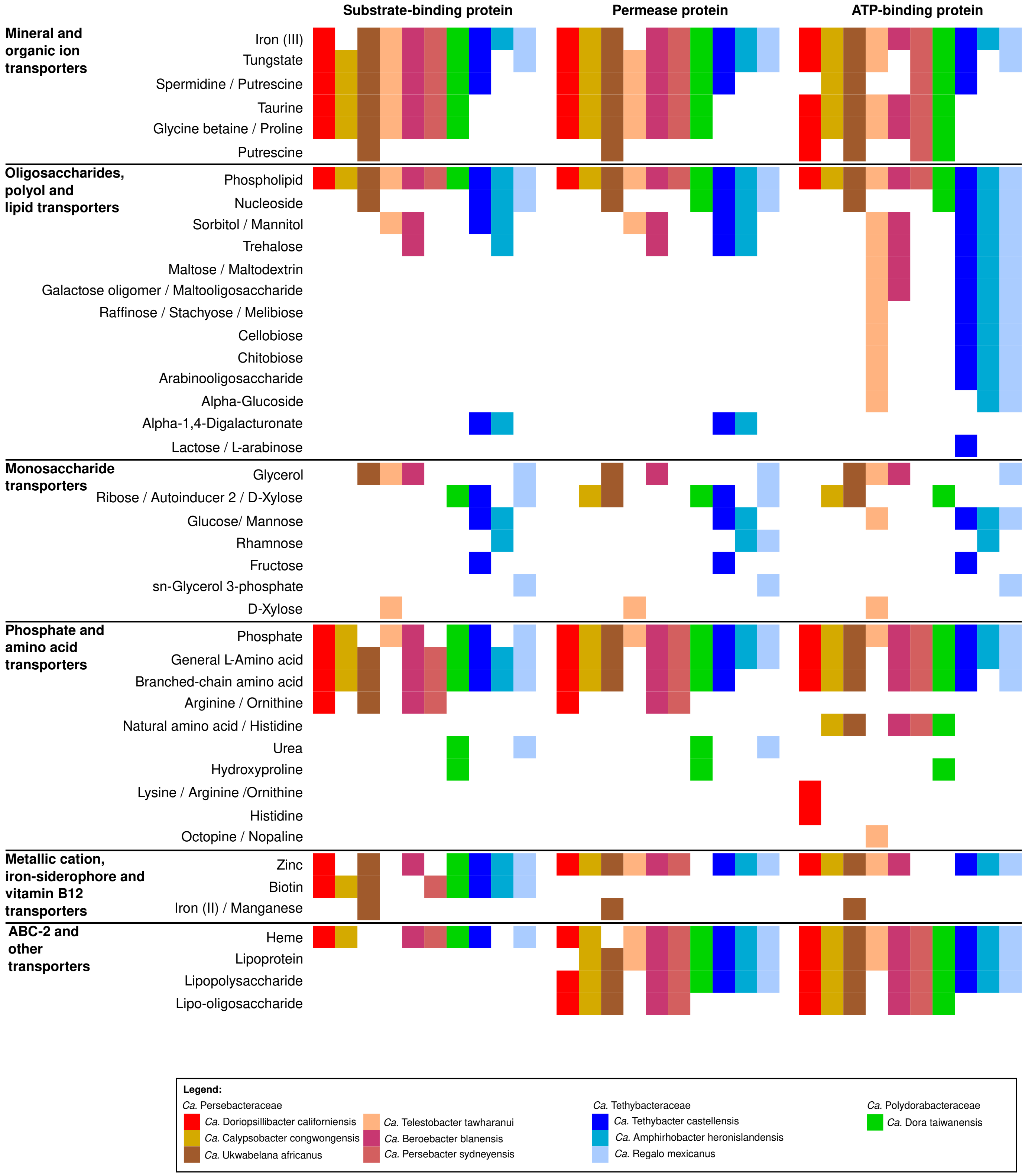

Supplementary Figure S6: Phylogenetic tree of 16S rDNA gene sequences from public datasets.
The tree was inferred using full length sequences of representatives of all Proteobacteria families, full length or nearly full length 16S rDNA sequences of all *Ca.* Tethybacterales MAGs from previous studies and also nearly full length sequence of Ca. Tethybacterales detected in public repositories in the study of Taylor et al. (2020). In addition, the tree contains partial 16S rDNA sequences from SRA and partial sequences from previous nudibranch microbiome studies that shared >92% sequence identity with *Ca.* D. californiensis (representing *Ca.* Persebacteraceae family cutoff). The squares represent the runs, are colored by the sample type, and include the number of reads in the given cluster (99% sequence similarity on 99% length). The SRA sample IDs are shortened to the last three numbers, if their ID starts with the same numbers and letters as the previous sample in their cluster.

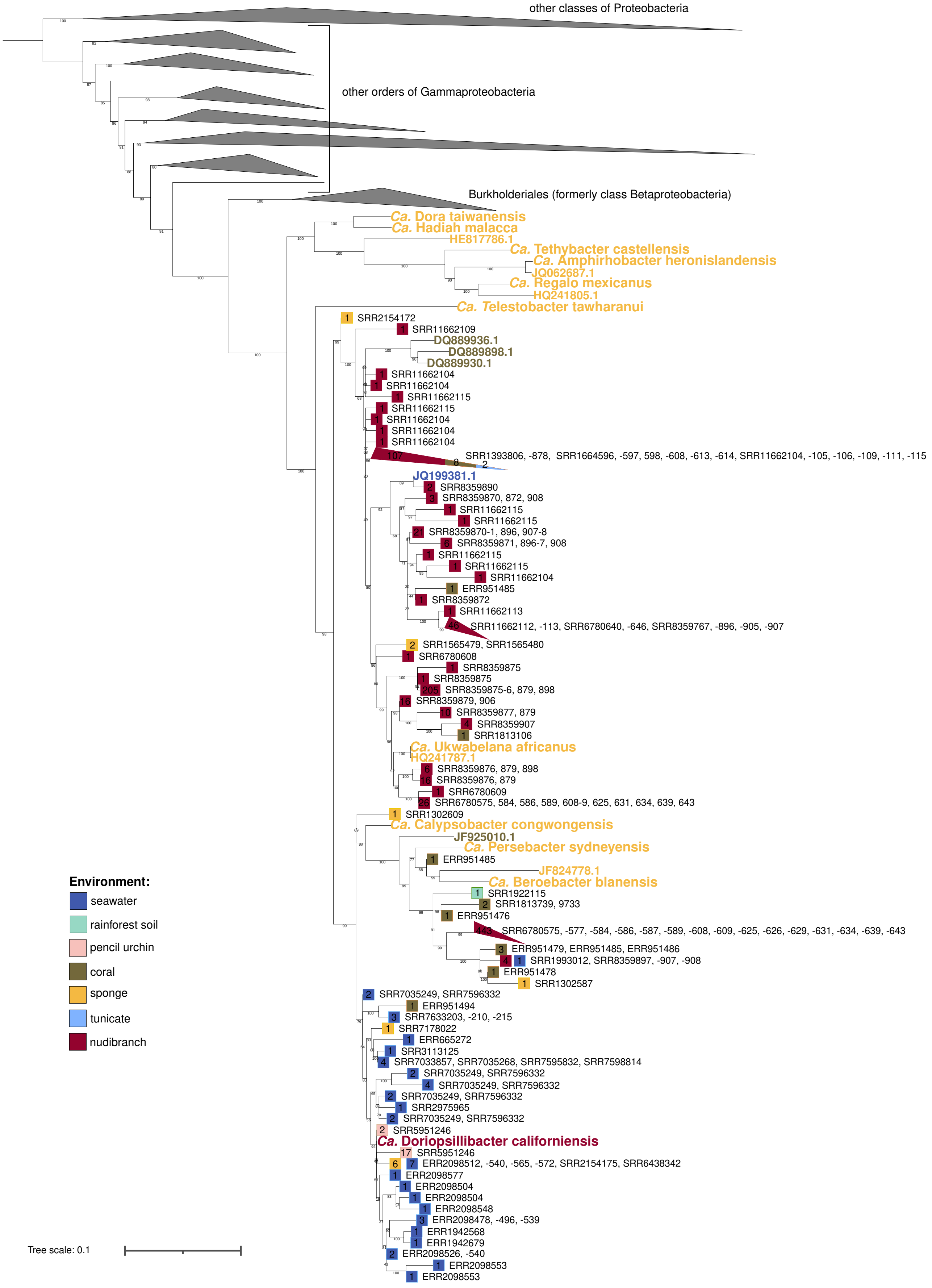

Supplementary Figure S7: Proportion of reads with >92% sequence similarity to *Ca.* D. californiensis in other nudibranchs. Proportion of reads that shared >92% sequence identity with *Ca.* D. californiensis (representing *Ca.* Persebacteraceae family cutoff) in previous studies on nudibranch microbiome (Cleary et al. and Abdelrahman et al.). The matched reads clustered more closely to *Ca.* Ukwabelana, *Ca.* Beroebacter or *Ca.* Persebacter than with *Ca.* Doriopsillibacter (Supplementary Figure S6).

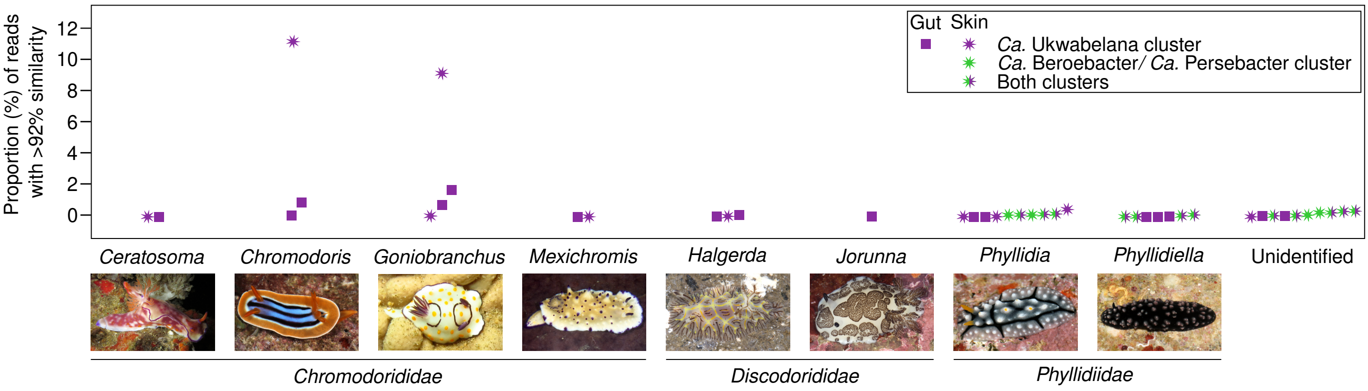

Supplementary Figure S8: Heatmap showing relative abundances of top ASVs across all 16S rDNA amplicon samples.
Only the ASVs with abundances >0.01% in at least two samples are shown. The samples and ASVs are clustered by the ward.D2 method.

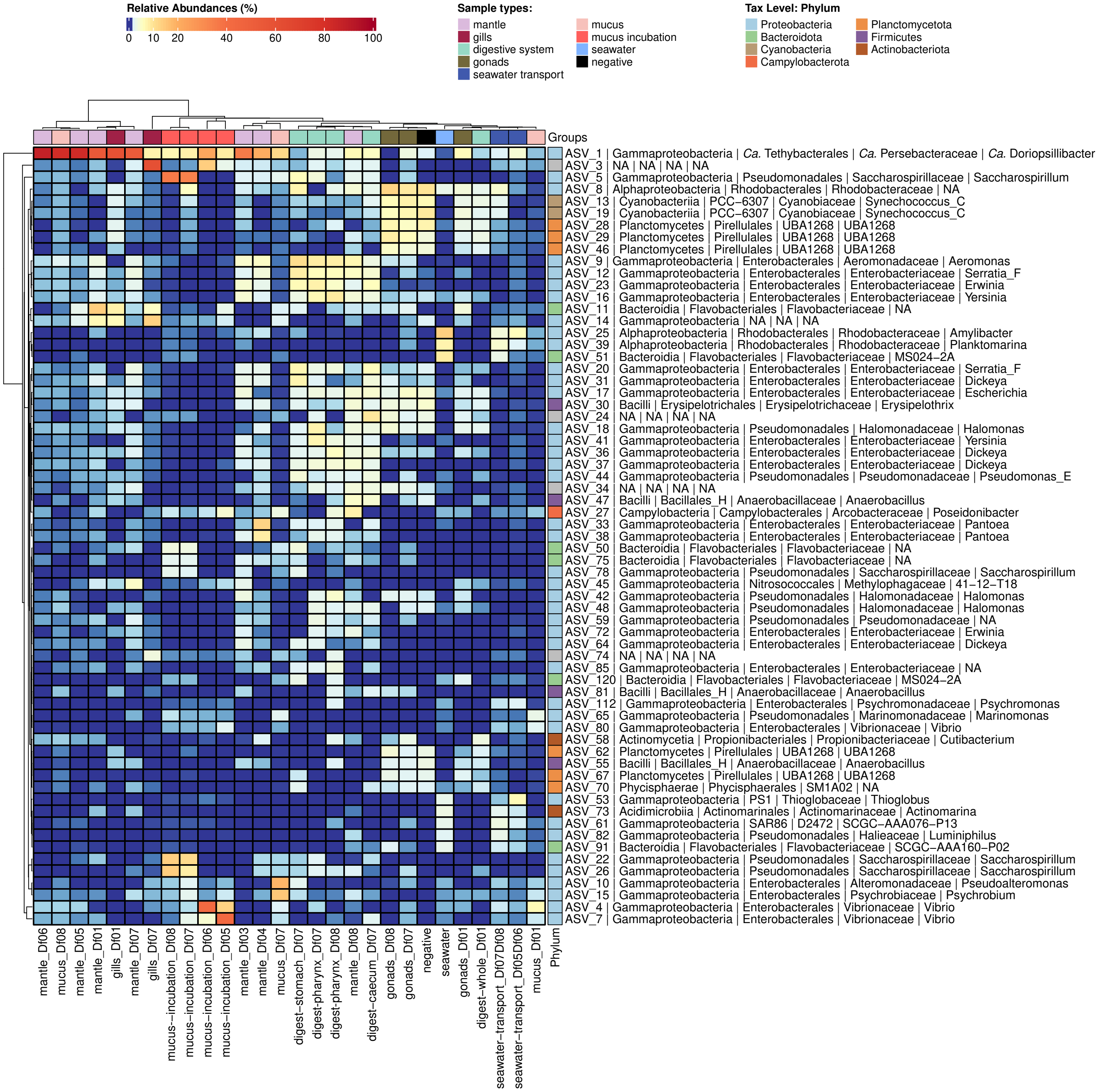

Supplementary Figure S9: Core microbiome of *D. fulva* mantle. a) Bacteria (ASVs) of the core mantle microbiome, as defined by different proportion thresholds. ASVs detected in all 7 nudibranchs at a proportion >0.01% are shown. Shades of green indicate the number of *D. fulva* specimens having a proportion of the listed ASV above the given threshold. **b)** Heatmap visualizing the relative abundances of the ASVs from *D. fulva* core mantle microbiome in different samples from the seven *D. fulva* specimens. The samples are ordered by decreasing relative proportion of ASV1 (*Ca.* Doriopsillibacter californiensis).

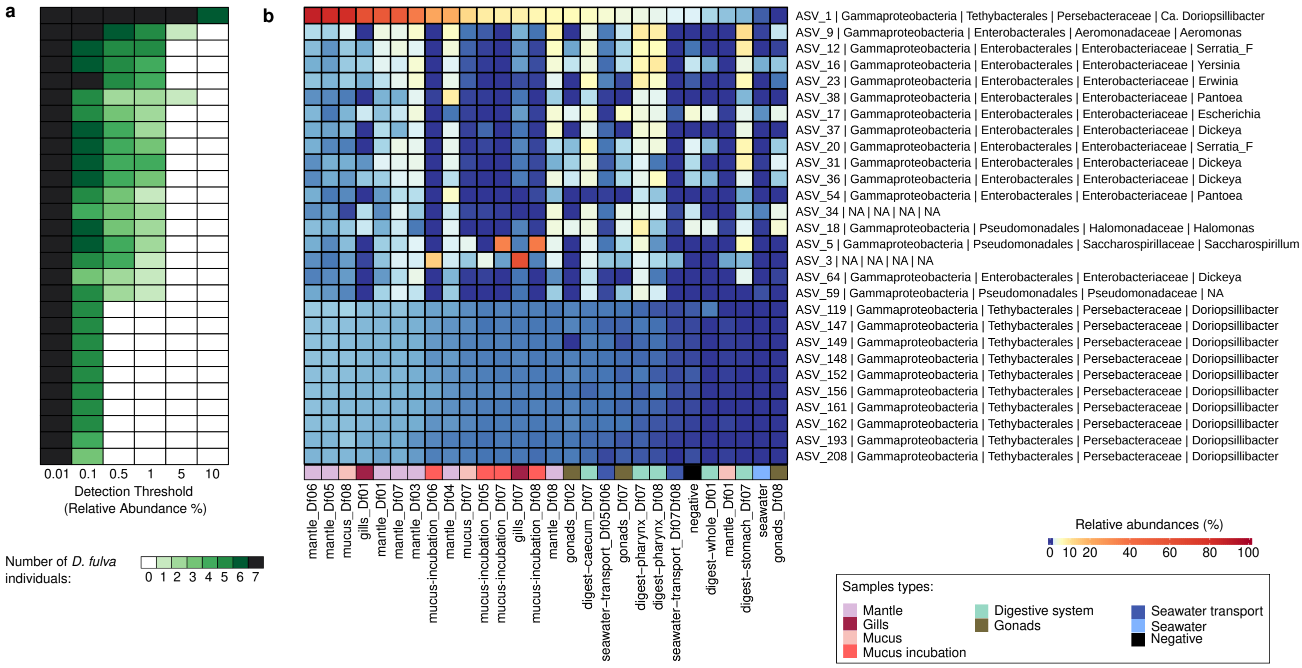

Supplementary Figure S10: ASVs determining the ordination of samples in the PCA anlaysis of nudibranch microbiome samples.
ASVs shown for the PC1 and PC2. The boxplots on the right show the proportion of these ASVs in the three groups of samples.

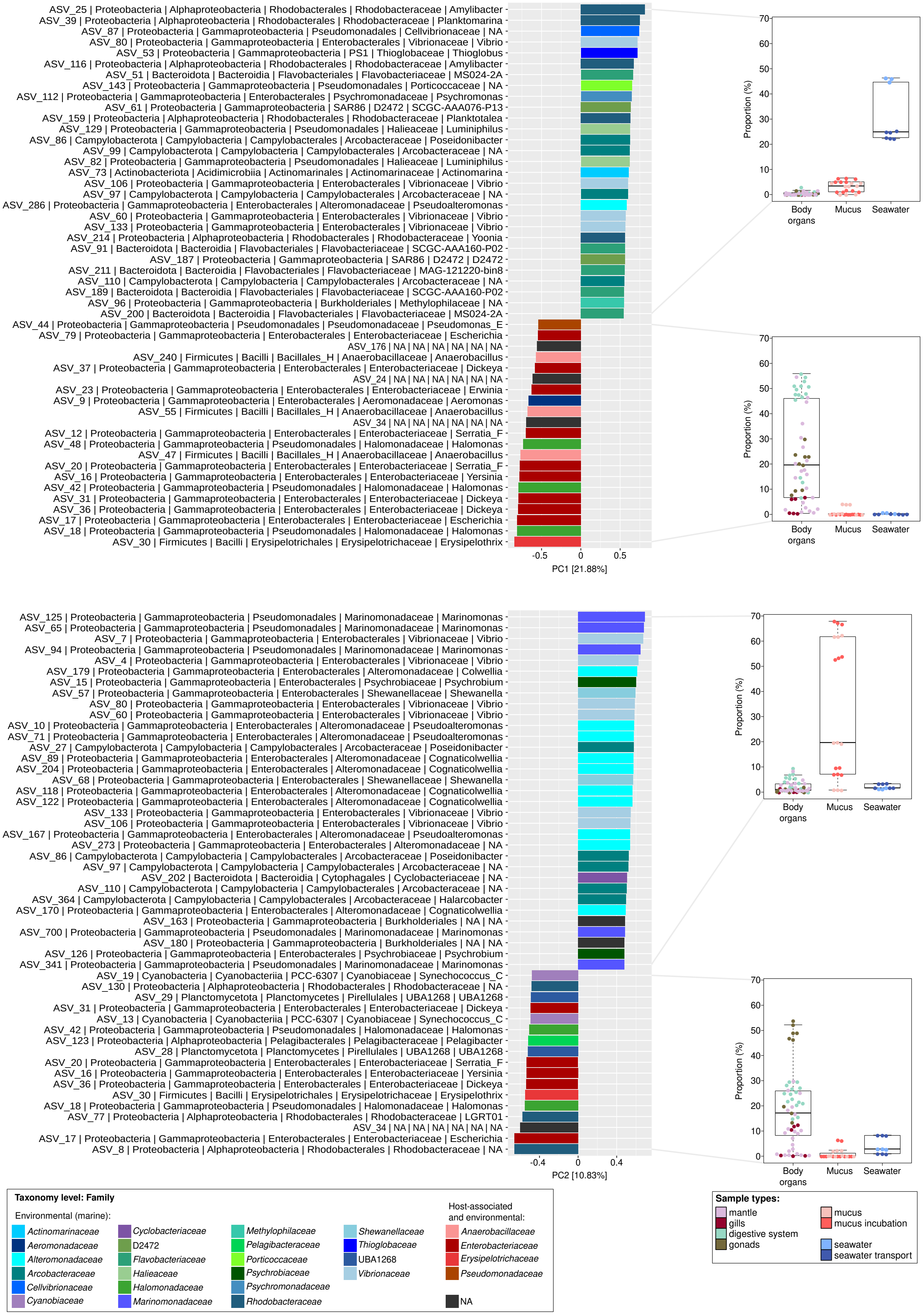

Supplementary Figure S11: Phylogenetic tree of full length 16S rDNA gene sequences from nudibranch skin and mucus obtained by Sanger sequencing.

Four hundred sequences were clustered by u-search into groups with 99% sequence similarity and classified by SILVA Alignment, Classification and Tree service. The ASV_3 in the tree refers to the abundant unclassified ASV detected also by sequencing of the V3-V4 16S rDNA regions on the Illumina platform in this study.

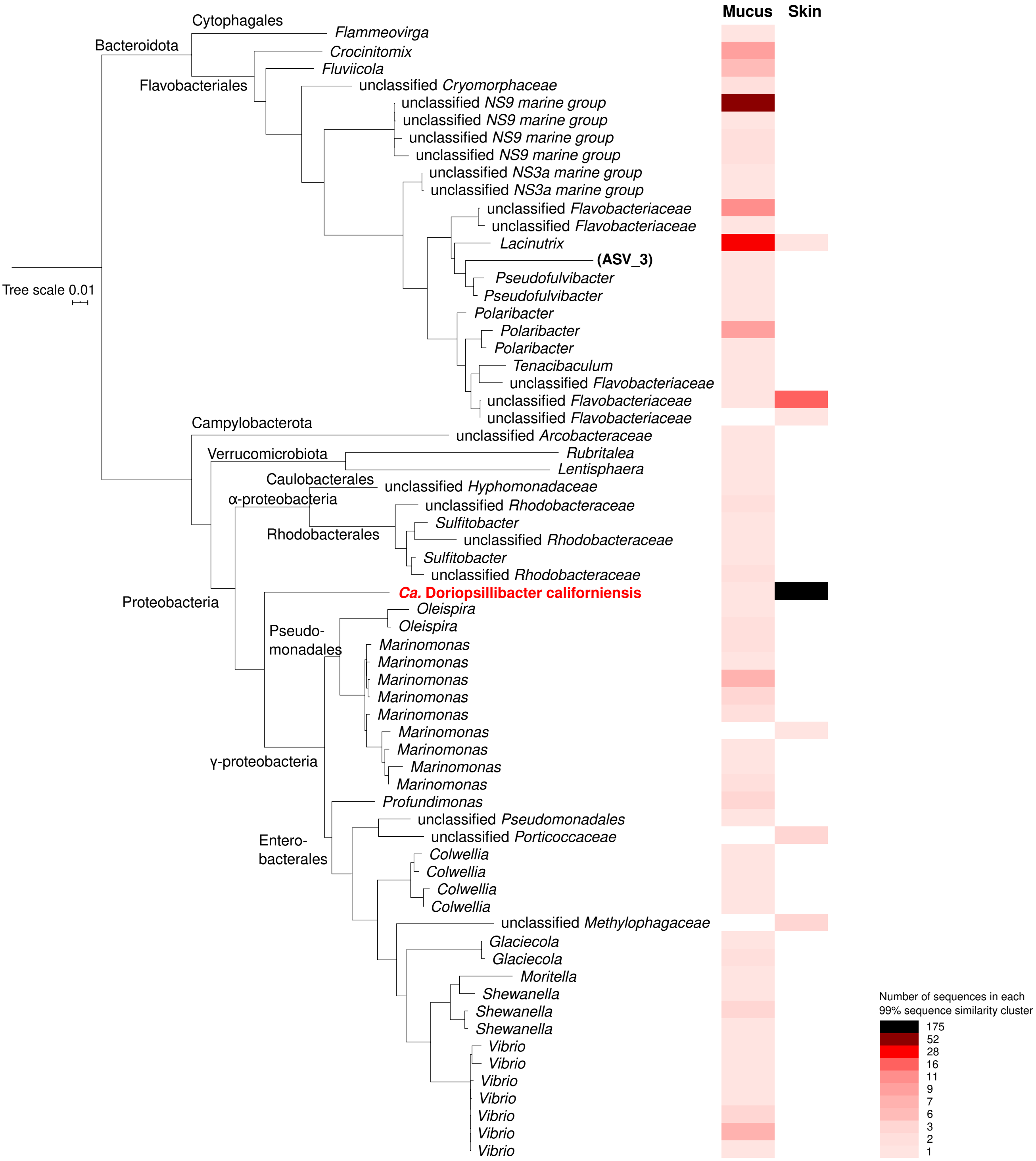

Supplementary Figure S12: *Ca.* D. californiensis betalactone gene cluster compared to the best antiSMASH matches.
The panel a) shows the most similar gene clusters found by antiSMASH v5.0.0 and v6.1.0. The matched genes are highlighted and the numbers above them show AA sequence similarity. In addition, the full genomes of the matched bacteria were searched for genes matching the *Ca.* D. californiensis betalactone gene cluster. The panel b) shows the highest sequence similarity found in the whole genome length and biosynthetic genes are labeled pink.

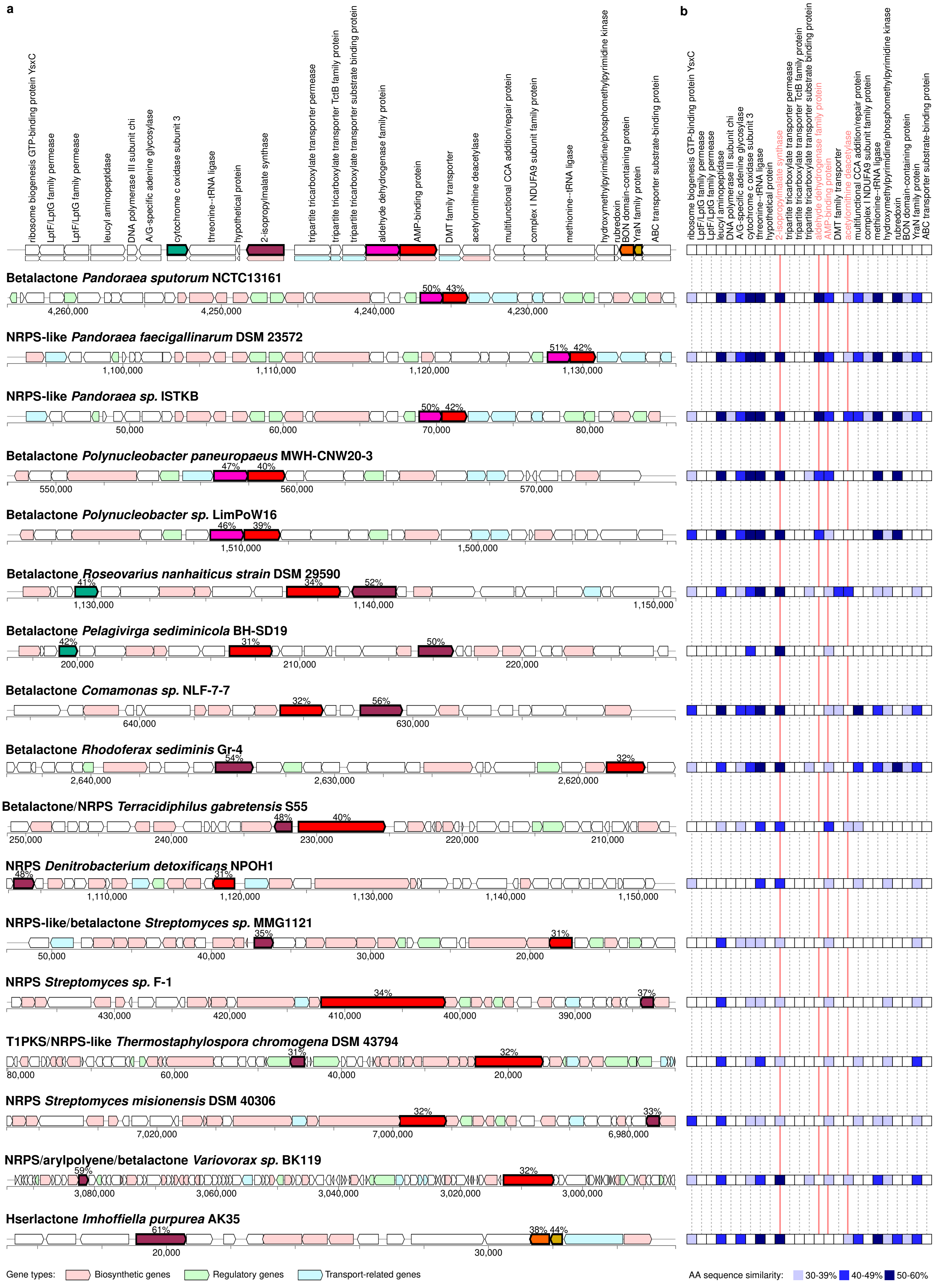

Supplementary Figure S13: Similarity of the genes in the *Ca.* D. californiensis betalactone gene cluster with the 'nr' database of NCBI. **a)** Amino acids sequence similarity of the genes in the *Ca.* D. californiensis betalactone (DCB) gene cluster with the 'nr' database of NCBI. The panel shows 17 genomes (GenBank assembly accession number included) with the most matches to the DCB, the matched genes were distributed across their whole genome length. The match with the highest AA sequence similarity for each gene is marked by a white star. Biosynthetic genes of the DCB are highlighted in pink and the core biosynthetic genes are marked by the orange rectangle. **b)** Visualization of the betalactone gene clusters detected in the 17 genomes by antiSMASH v6.1.0. The biosynthetic genes are highlighted by pink and include gene function descriptions. Only three betalactone gene clusters had some AA sequence similarity with the AMP-dependent synthetase and ligase from the DCB gene cluster (similarity 30-33%, as indicated by the numbers inside the matched genes).

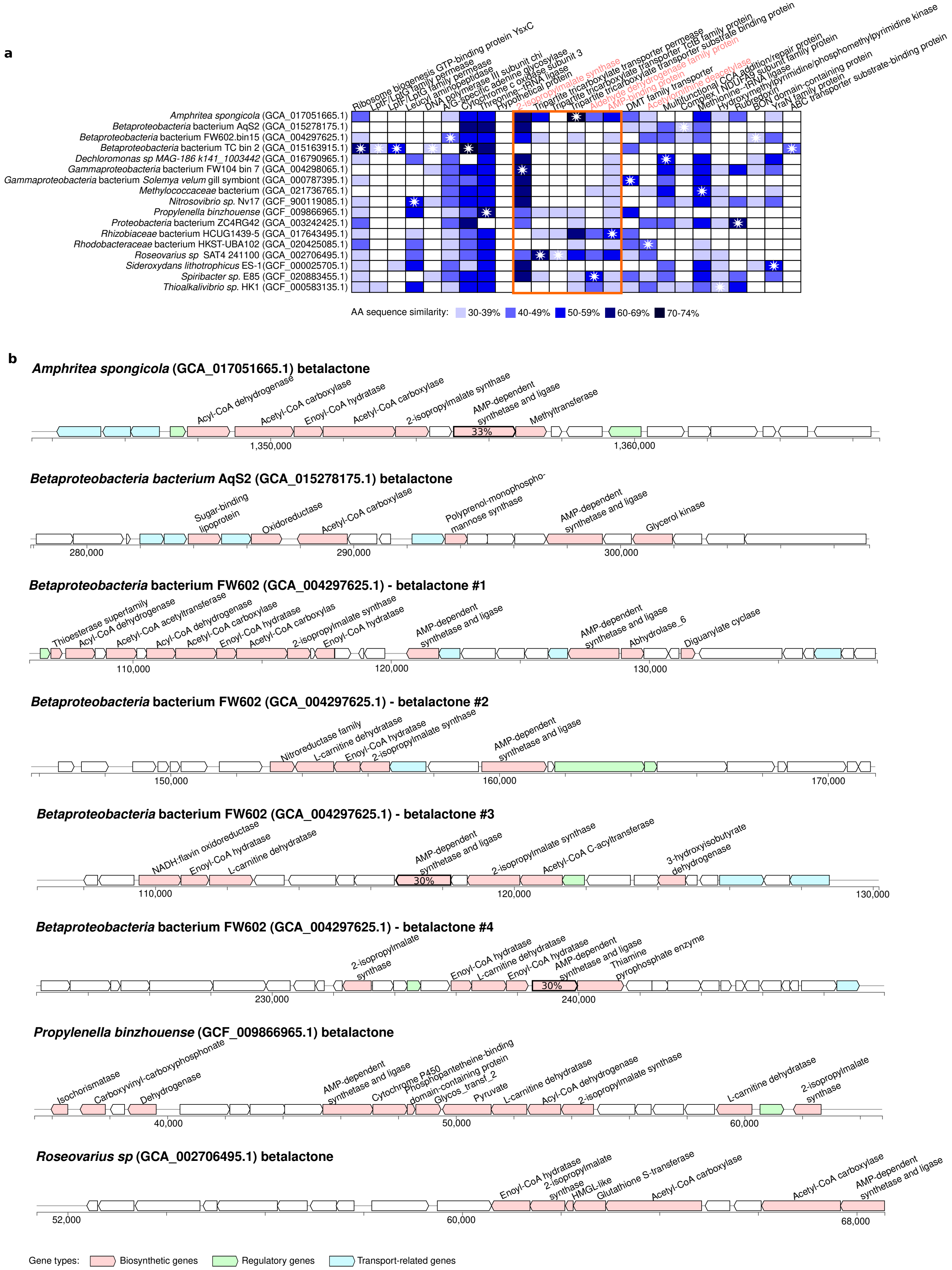

Supplementary Figure S14: Similarity of the genes in the *Ca.* D. californiensis betalactone gene cluster with other members of the *Ca.* Tethybacterales order. a) Amino acid sequence similarity of the genes in the *Ca.* D. californiensis betalactone (DCB) gene cluster with other members of the *Ca.* Tethybacterales order. The matched genes were distributed across their whole genome length, they were not organized in any biosynthetic gene clusters. Biosynthetic genes of the DCB are highlighted in pink and the core biosynthetic genes are marked by the orange rectangle. **b)** Visualization of the different biosynthetic gene clusters detected in other members of the *Ca.* Tethybacterales order by antiSMASH v6.1.0. The biosynthetic genes are highlighted by pink and include gene function description. None of the genes in these clusters shared a AA similarity >30% on the 60% gene length with the DCB genes.

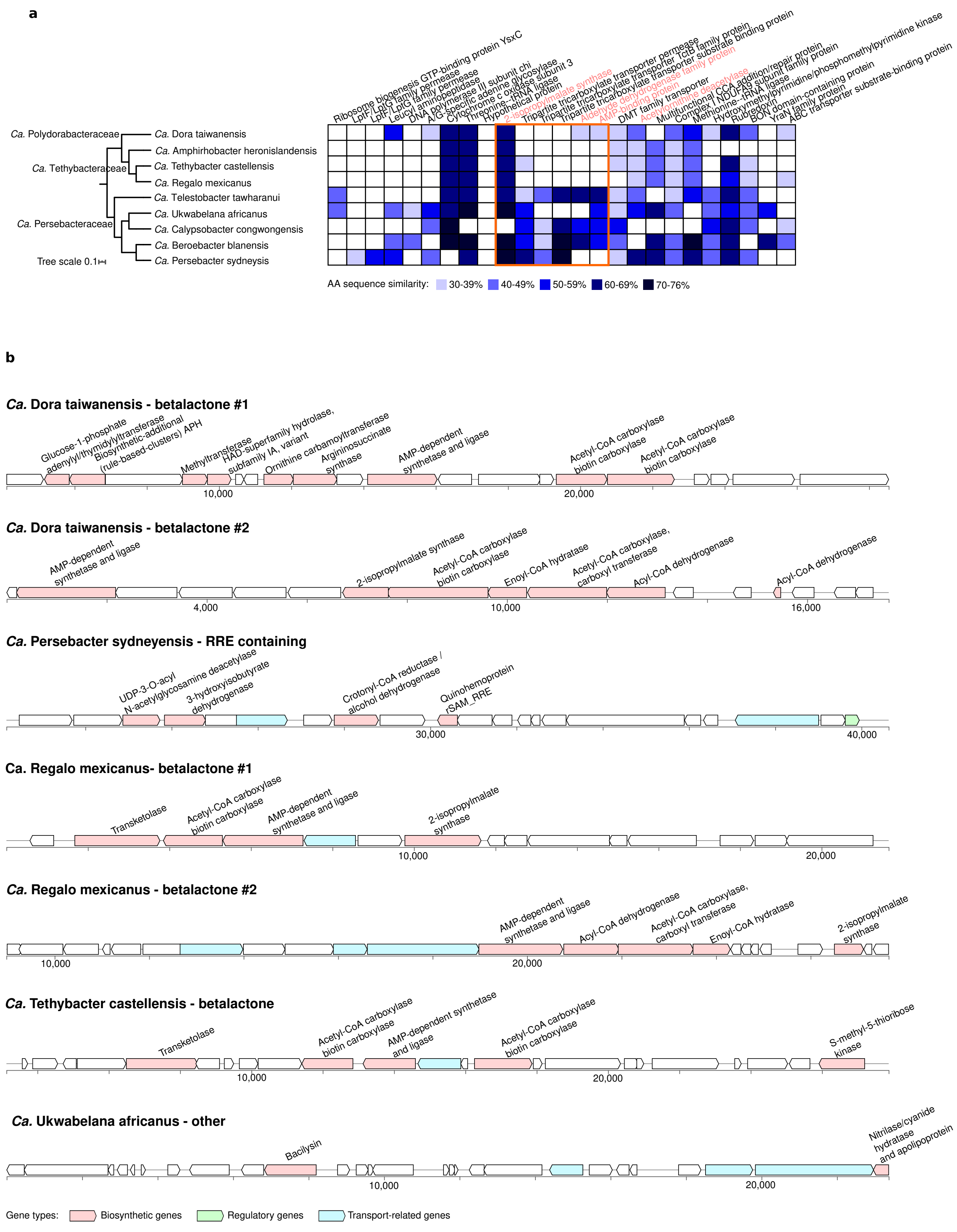

Supplementary Figure S15: Biosynthetic pathway comparison to known betalactone natural product pathways.
a) Structures of five exemplary betalactone natural products. Current understanding of the biosynthesis pathways to **b)** cystargolide B (Wolf et al., 2017) and **c)** belactosin C (Engelbrecht et al., 2022). **d)** Proposed biosynthetic pathway for DCB. Our DCB biosynthetic gene cluster encodes three proteins DcbA (green), DcbF (red) and DcbH (orange) that share comparable functions and protein homology as that observed in belactosin and cystargolide pathways. Color codes are used to show the position of each of these enzymes in their respective pathways. **e)** The genes within the DCB cluster.

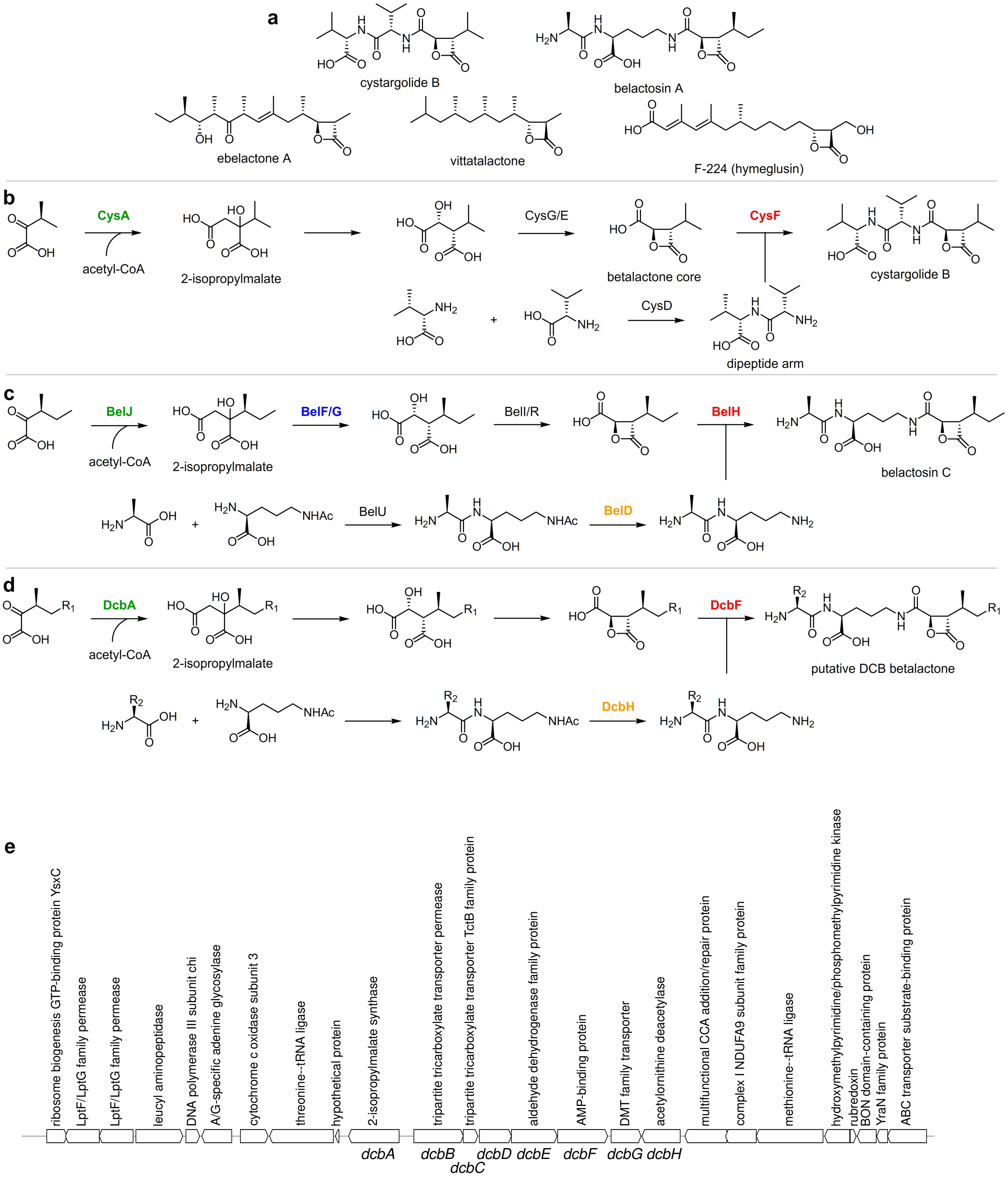

Supplementary Figure S16: NMR analysis of extracts from *D. fulva* nudibranchs.
Microscaled NMR was used to guide the extraction process. 1H-NMR spectrum of the Df03 specimen with **a)** CH2Cl2, **b)** ethyl acetate, **c)** acetone and **d)** methanol. Red spheres and blue squares are used to identify the peaks that match the assignment to the predicted DCB betalactone. Insets are provided to enhance the visualization of the two assigned peaks.

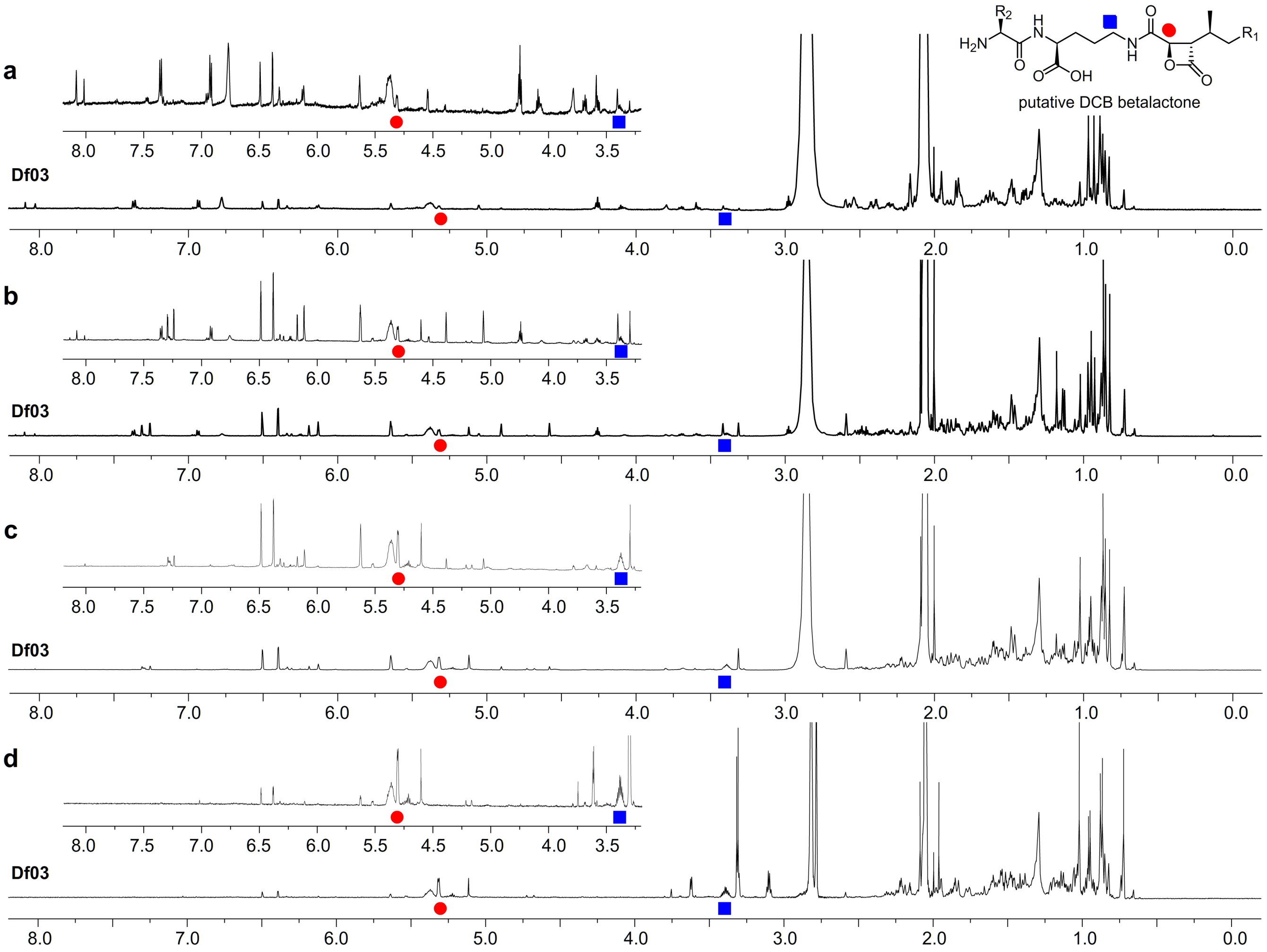

Supplementary Figure S17: Quantification of the *dcbD* expression in the CRAGE strains and nudibranch samples.

a) Table showing the cycle thresholds (Ct) of the amplification curves of the qPCR reactions targeting the *dcbD* gene in the cDNA of the seven CRAGE strains cultured with IPTG (0.01mM), compared to the DNA extracted from the same CRAGE strains. The experiment involved negative controls of DNA extracted from the wild type strains, which showed no amplification. *Aeromonas piscicola* had the largest ΔCt difference between the copies of the *dcbD* gene in cDNA compared to DNA (marked by an asterisk), thus it was selected for further experiments. **b)** The sequences of *dcbD* primers targeting codon-optimized DCB in the CRAGE strains were compatible with the original DCB sequence of *Ca.* D. californiensis, which permitted us to use DNA extracted from DCB+ CRAGE strains as positive controls in qPCR assays for detection of *dcbD* expression in *D. fulva* nudibranch samples. Although the bacterial fraction in nudibranch samples was enriched by filtration through 5 µm pores, it is very likely that some nudibranch cells were still present in the filtered sample and finally comprised the largest portion of the total extracted RNA. The nudibranch host RNA contamination is obvious from the qPCR results, which indicated that the cDNA from nudibranch skin samples contained at least 100x less *dcbD* copies than the DNA sample, and that *dcbD* formed approximately 0.0001% of the total cDNA from the skin sample, which is about 560,000-920,000x less than the proportion of *Ca.* D. californiensis in the nudibranch Df07 mantle microbiome estimated by the 16S rDNA-based methods. Despite large host RNA contamination, we have demonstrated *dcbD* expression in the skin samples and its absence in the nudibranch internal organs. The expression of *dcbD* and the *Ca.* D. californiensis housekeeping gene *ihfB* was close to the qPCR detection limit, however, the melting curve profiles of *dcbD* in cDNA skin samples matched the DCB+ CRAGE strains positive control and the DNA extracted from Df07 skin sample, which confirms the correct amplicon size. The figures show the amplification curves on the left and the melting curves on the right. The experiments were performed with 0.5 ng/ul of extracted DNA or synthesized cDNA.

a

	cDNA	DNA	ΔCt	
Aeromonas piscicola	15.08 ± 0.13	16.77 ± 0.10	1.70	*
Aeromonas salmonicida subsp. pectinolytica 34mel	16.68 ± 0.12	16.99 ± 0.08	0.31	
Erwinia oleae	19.45 ± 0.16	18.86 ± 0.05	-0.60	
Pantoea agglomerans	16.81 ± 0.05	17.85 ± 0.24	1.04	
Pseudomonas putida KT2440	18.07 ± 0.01	18.55 ± 0.29	0.48	
Yersinia aldovae	22.42 ± 0.19	17.79 ± 1.10	-4.63	
Yersinia mollaretii	19.41 ± 0.10	16.26 ± 1.77	-3.15	

b

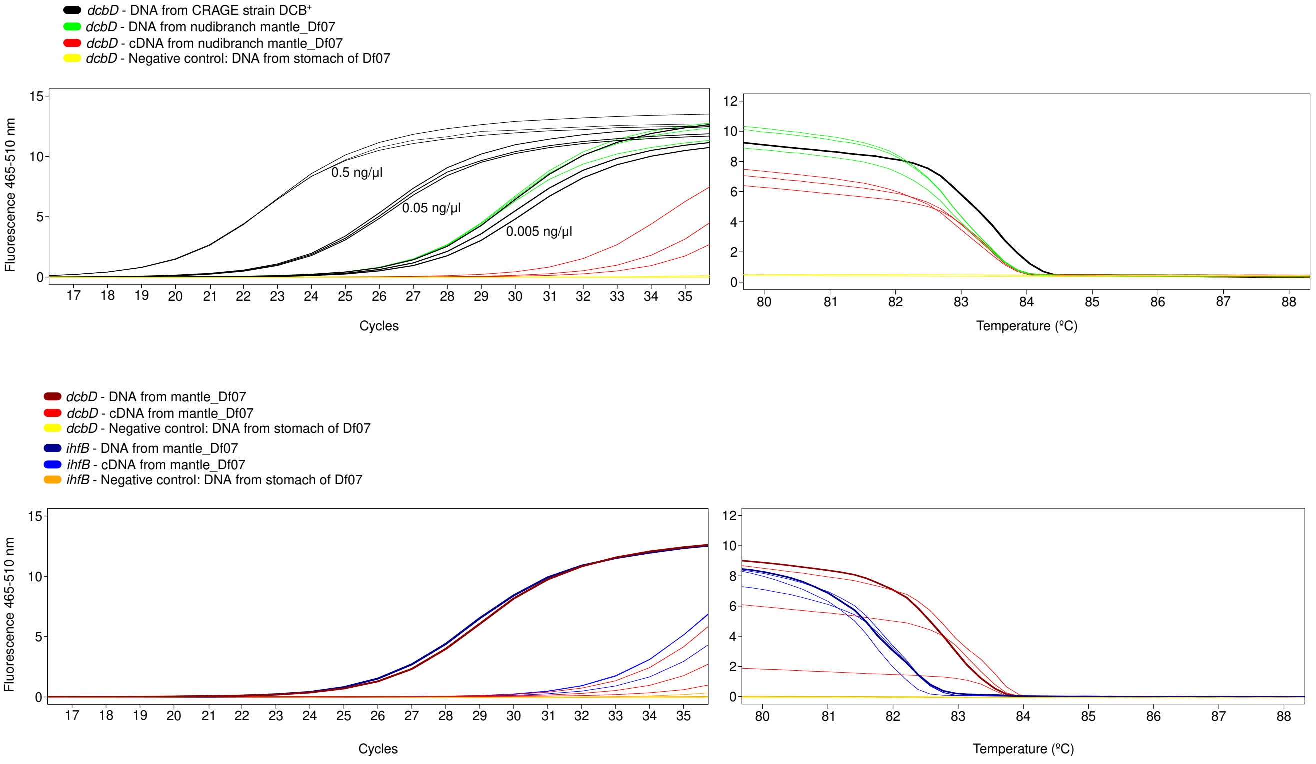

Supplementary Figure S18: Testing of the CRAGE strain *Aeromonas piscicola* for betalactone expression induced by IPTG. Plots showing cycle threshold (Ct) values of the samples *A. piscicola* DCB⁺ and the control strain lacking the DCB insert (containing only the CRAGE landing pad) cultured in the M9 media. The first samples were taken three hours after transferring the culture from LB to M9. Afterwards, different concentrations of IPTG were added and the samples were cultured for 3 days. **a)** The ΔCt values represent the difference between the average Ct of all six core DCB genes and the *A. piscicola* housekeeping genes *ihfA* and i*hfB*, and the ΔΔCt values are the difference between Ct of the different IPTG concentration compared to the 3 hours culture. **b)** The samples from *A. piscicola* DCB⁺ 3 days culture with 0.01mM IPTG were compared to negative controls of *A. piscicola* DCB⁺ 3 days culture and *A. piscicola* lacking the DCB insert cultured for 3 days under the same conditions. The experiments were performed using RNA extracted in triplicates, using 5 µl of 0.1 ng/µl cDNA in each reaction. **c)** List of primers used in these qPCR experiments.

a

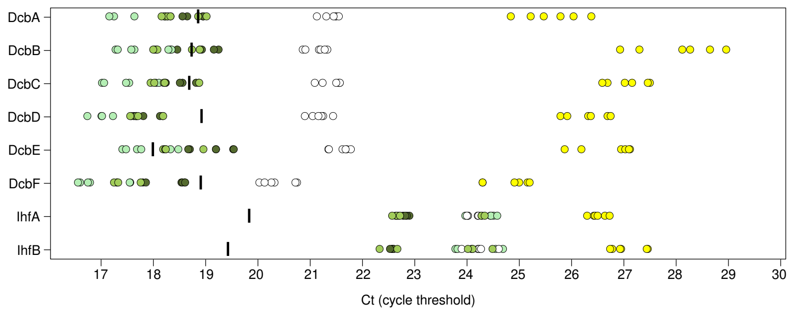

	DCB	Ihf	ΔCt	ΔΔCt
First 3 hours in M9	26.42 ± 1.15	26.77 ± 0.37	0.35 ± 1.10	
Cultured in M9 with 0 mM IPTG	21.17 ± 0.44	24.19 ± 0.18	3.02 ± 0.43	2.67
Cultured in M9 with 0.01 mM IPTG	17.49 ± 0.53	24.24 ± 0.32	6.75 ± 0.53	6.39
Cultured in M9 with 0.1 mM IPTG	18.10 ± 0.50	23.34 ± 0.87	5.18 ± 2.08	4.82
Cultured in M9 with 1 mM IPTG	18.59 ± 0.48	22.67 ± 0.13	4.12 ± 0.50	3.77

b

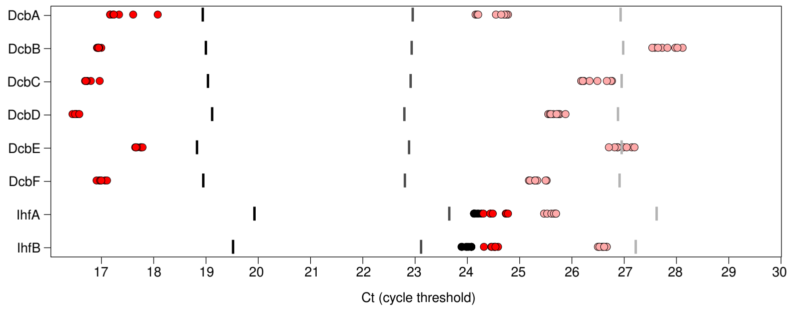

	DCB	Ihf	ΔCt	ΔΔCt
No insert, cultured for 3 days		24.08 ± 0.11		
DCB⁺, cultured for 3 hours	26.11 ± 1.12	26.13 ± 0.50	0.02 ± 1.19	
DCB⁺, cultured for 3 days	17.03 ± 0.42	24.53 ± 0.15	7.49 ± 0.44	7.47

Average Ct of DNA extracted from *A. piscicola* DCB⁺: | 0.1 ng/µ | 0.01 ng/µl | 0.001 ng/µl

c

Primer	Sequence (5′-3′)
DcbA-F	TTCGACACAACGCTACGTGA
DcbA-R	ACAGCCCTTGCCAGTACTTC
DcbB-F	GGGATTGTGGTTGGACTCGT
DcbB-R	TTGGCCCACCAACAGTGAAT
DcbC-F	TTATGGCGGTTTGGGCTTCT
DcbC-R	CAACGGCACCTGTCACAATG
DcbD-F	GCAAAAAGGGTGGAATGCGT
DcbD-R	CATTCACCGTCAGTTTGCCG
DcbE-F	GTAGCGGCTCTCACTCCTTC
DcbE-R	TGACCAACATCGGCTGTCTC
DcbF-F	GCCGTTTTTCCTGAGCTTGG
DcbF-R	CATATCGCAAAGCGCGTCAA
IhfA-F	ATCTTCGCCCGTCTTAGGGT
IhfA-R	ACCAAAGCCGACATTGCAGA
IhfB-F	TCATCGAACAATTGGCTGCG
IhB-R	CCCGGTCAGTTCAACCTTGT

Supplementary Figure S19: Feature-based molecular network identifying metabolite production in the *A. piscicola* DCB⁺ CRAGE strain.
To identify potential betalactone natural products, we performed feature based molecular networking to compare metabolite production between the heterologous host and the empty vector control. No putative betalactones were identified, but we did observe differences in the production of phophoethanolamines and cyclo dipeptides between the two strains.
Green = *A. piscicola* DCB⁺ 3 days culture, Yellow = *A. piscicola* LP 3 days culture, purple = QC mix.
A. Differences in production of phosphoethanolamines.
B. Increased production of Cyclo-peptides by *A. piscicola* DCB⁺ host.

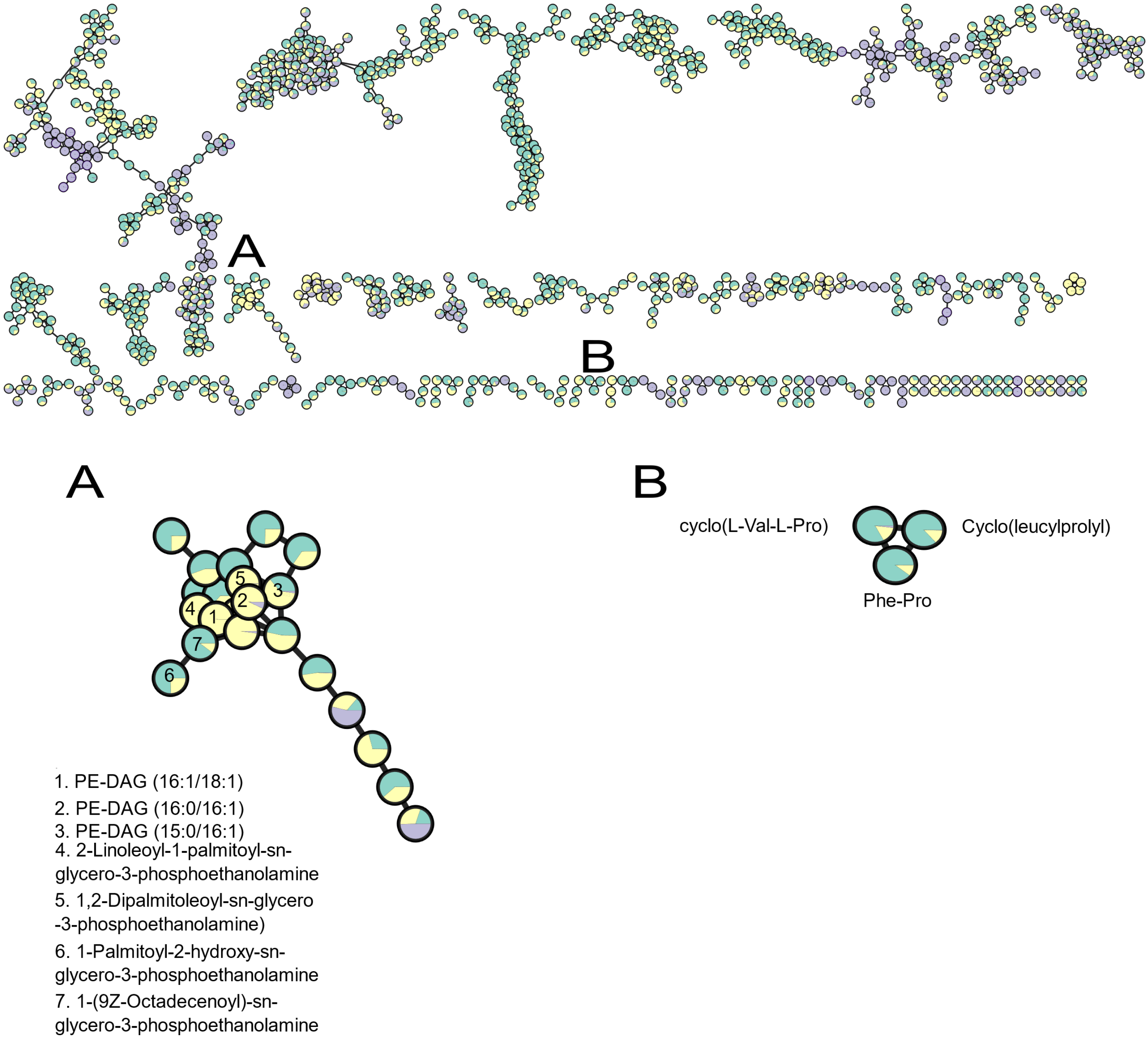

Supplement: Supplementary file 2 — Additional file 1: Supplementary Figure S1. Photorhabdus luminescens incubated with the KC-12 probe during 70 h. Supplementary Note 1. Taxonomic Appendix. Supplementary Figure S2. Hybridization of cells from nudibranch skin, gut, gills and gonads. Supplementary Figure S3. KEGG modules of Ca. D. californiensis and medium quality Ca. Tethybacterales MAGs from sponges. Supplementary Figure S4. Sulfur and nitrogen metabolism of Ca. D. californiensis and medium quality Ca. Tethybacterales MAGs from sponges. Supplementary Figure S5. ABC transporters of Ca. D. californiensis and medium quality Ca. Tethybacterales MAGs from sponges. Supplementary Figure S6. Phylogenetic tree of 16S rDNA gene sequences from public datasets. Supplementary Figure S7. Proportion of reads with >92% sequence similarity to Ca. D. californiensis in other nudibranchs. Supplementary Figure S8. Heatmap showing relative abundances of top ASVs across all 16S rDNA amplicon samples. Supplementary Figure S9. Core microbiome of D. fulva mantle. Supplementary Figure S10. ASVs determining the ordination of samples in the PCA anlaysis of nudibranch microbiome samples. Supplementary Figure S11. Phylogenetic tree of full length 16S rDNA gene sequences from nudibranch skin and mucus obtained by Sanger sequencing. Supplementary Figure S12. Ca. D. californiensis betalactone gene cluster compared to the best antiSMASH matches. Supplementary Figure S13. Similarity of the genes in the Ca. D. californiensis betalactone gene cluster with the 'nr' database of NCBI. Supplementary Figure S14. Similarity of the genes in the Ca. D. californiensis betalactone gene cluster with other members of the Ca. Tethybacterales order. Supplementary Figure S15. Biosynthetic pathway comparison to known betalactone natural product pathways. Supplementary Figure S16. NMR analysis of extracts from D. fulva nudibranchs. Supplementary Figure S17. Quantification of the dcbD expression in the CRAGE strains and nudibranch samples. Supplementary F [file 40168_2023_1560_MOESM1_ESM.pdf]
